# AutoPeptideML: a study on how to build more trustworthy peptide bioactivity predictors

**DOI:** 10.1093/bioinformatics/btae555

**Published:** 2024-09-18

**Authors:** Raúl Fernández-Díaz, Rodrigo Cossio-Pérez, Clement Agoni, Hoang Thanh Lam, Vanessa Lopez, Denis C Shields

**Affiliations:** IBM Research, Dublin, Dublin D15 HN66, Ireland; School of Medicine, University College Dublin, Dublin D04 C1P1, Ireland; Conway Institute of Biomolecular and Biomedical Science, University College Dublin, Dublin D04 C1P, Ireland; The SFI Centre for Research Training in Genomics Data Science, Ireland; School of Medicine, University College Dublin, Dublin D04 C1P1, Ireland; Conway Institute of Biomolecular and Biomedical Science, University College Dublin, Dublin D04 C1P, Ireland; Department of Science and Technology, National University of Quilmes, Bernal B1876, Provincia de Buenos Aires, Argentina; School of Medicine, University College Dublin, Dublin D04 C1P1, Ireland; Conway Institute of Biomolecular and Biomedical Science, University College Dublin, Dublin D04 C1P, Ireland; Discipline of Pharmaceutical Sciences, School of Health Sciences, University of KwaZulu-Natal, Durban 4000, South Africa; IBM Research, Dublin, Dublin D15 HN66, Ireland; IBM Research, Dublin, Dublin D15 HN66, Ireland; School of Medicine, University College Dublin, Dublin D04 C1P1, Ireland; Conway Institute of Biomolecular and Biomedical Science, University College Dublin, Dublin D04 C1P, Ireland

## Abstract

**Motivation:**

Automated machine learning (AutoML) solutions can bridge the gap between new computational advances and their real-world applications by enabling experimental scientists to build their own custom models. We examine different steps in the development life-cycle of peptide bioactivity binary predictors and identify key steps where automation cannot only result in a more accessible method, but also more robust and interpretable evaluation leading to more trustworthy models.

**Results:**

We present a new automated method for drawing negative peptides that achieves better balance between specificity and generalization than current alternatives. We study the effect of homology-based partitioning for generating the training and testing data subsets and demonstrate that model performance is overestimated when no such homology correction is used, which indicates that prior studies may have overestimated their performance when applied to new peptide sequences. We also conduct a systematic analysis of different protein language models as peptide representation methods and find that they can serve as better descriptors than a naive alternative, but that there is no significant difference across models with different sizes or algorithms. Finally, we demonstrate that an ensemble of optimized traditional machine learning algorithms can compete with more complex neural network models, while being more computationally efficient. We integrate these findings into AutoPeptideML, an easy-to-use AutoML tool to allow researchers without a computational background to build new predictive models for peptide bioactivity in a matter of minutes.

**Availability and implementation:**

Source code, documentation, and data are available at https://github.com/IBM/AutoPeptideML and a dedicated web-server at http://peptide.ucd.ie/AutoPeptideML. A static version of the software to ensure the reproduction of the results is available at https://zenodo.org/records/13363975.

## 1 Introduction

Peptides are short amino acid chains with 3–50 residues with a great variety of therapeutical properties. They have gained a lot of attention from the pharmaceutical and food industries, as their versatility makes them excellent candidates for drug or nutraceutical discovery (Wang *et al.* 2022). In this context, there is a growing demand for predictive models that can accelerate the discovery or design of peptides targeting new properties or bioactivities (Attique *et al.* 2020).

Novel developments in machine learning algorithms have offered new models for predicting protein structure (Lin *et al.* 2022) or different molecular properties (Dara *et al.* 2022). Despite these advancements, developing and evaluating new models is still an arduous process that requires both domain expertise and technical skills (Attique *et al.* 2020). Thus, most predictive models target broad and general applications, while solutions for more narrow use cases, like specific peptide bioactivities, remain underdeveloped (Attique *et al.* 2020).

**Figure 1.**
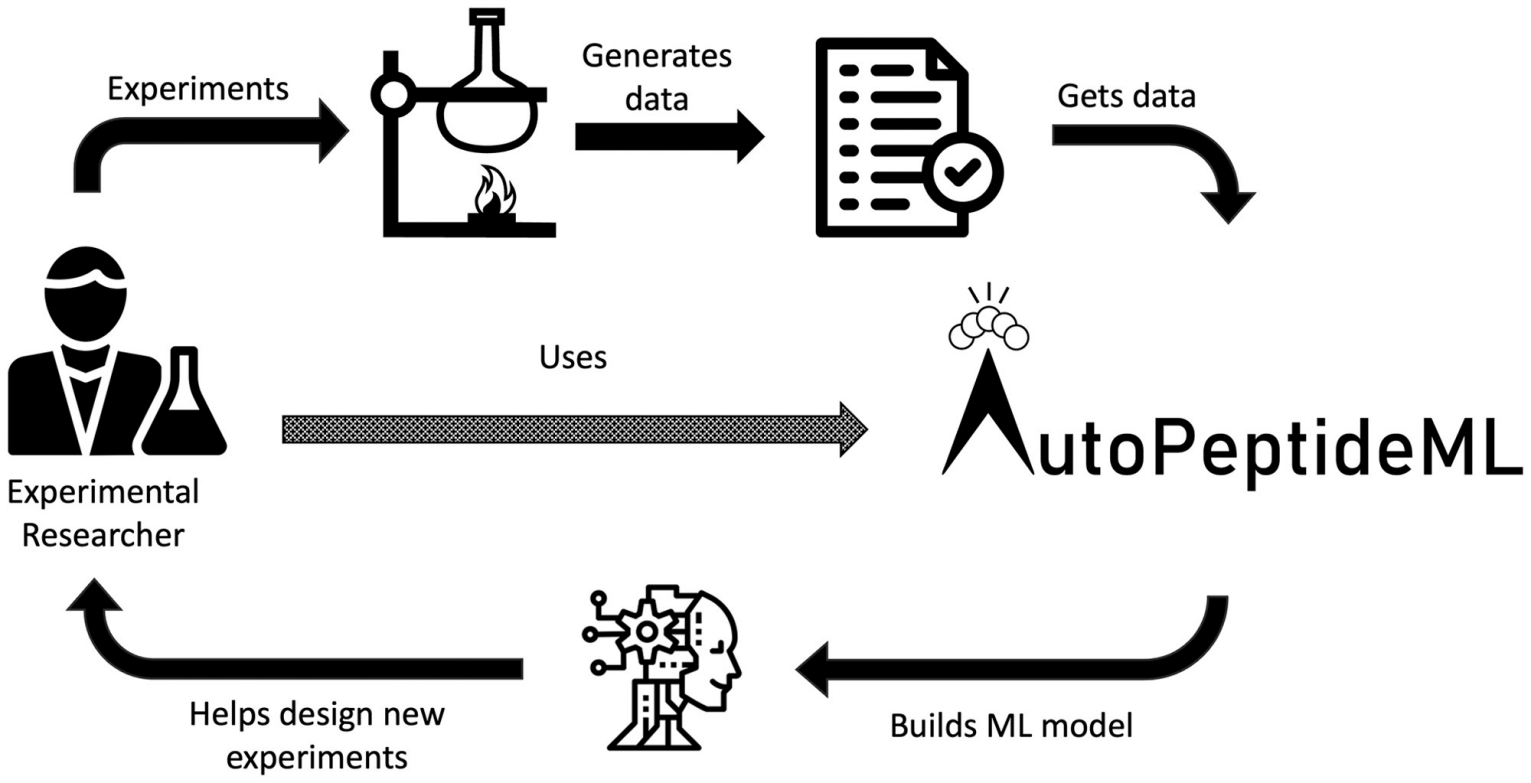
Integration of AutoPeptideML in an experimental workflow. AutoPeptideML allows experimental researchers to build new custom models from their data. These models can then be used to propose new experiments. The data generated from these experiments can, in turn, be used to generate a new improved model. This leads to a feedback loop where the experimentation is guided towards more relevant peptides with each iteration.

Here, we propose building an automated machine learning (AutoML) system to automate the development of custom bioactivity predictors. There are several benefits that the introduction of such a system would provide. First, AutoML solutions can reduce the time required for model development from weeks to hours (He *et al.* 2021). Second, they help democratize machine learning by enabling researchers without a computational background to build effective models (He *et al.* 2021). Third, they help to ensure that best practices are followed, which can lead to increased trust in ML predictors within the field (Amirian *et al.* 2021). Fourth, they can greatly simplify and accelerate current strategies that require not only strong computational skills, but also very tedious experimentation through extensive trial-and-error (Attique *et al.* 2020). Overall, an AutoML tool for building peptide bioactivity predictors will allow experimental researchers to seamlessly introduce advanced modelling techniques into their experimental workflows in a matter of hours (see Fig. 1).

A review of the existing literature revealed five key steps in the development of a peptide bioactivity predictor that could be benefited by this automation: (i) data gathering for negative peptides, (ii) dataset partitioning, (iii) computational representation of the peptides, (iv) model training and hyperparameter optimization, and (v) reporting of model evaluation.

1) **Data gathering for the negative peptides**. Binary classifiers require both positive and negative examples. Finding positive examples for peptide bioactivity is relatively simple as there is prior literature describing the function and role of a multitude of peptides (Quiroz *et al.* 2021). However, there are few repositories enumerating peptides that do not present a certain function or property (Attique *et al.* 2020). Further, there is no consensus in the literature as to how negative peptides should be chosen: some works opt for choosing fragments of proteins (Manavalan *et al.* 2019, Bin *et al.* 2020, Agrawal *et al.* 2021) others look for actual peptides (Agrawal *et al.* 2021, Pinacho-Castellanos *et al.* 2021, Charoenkwan *et al.* 2022c, Pang *et al.* 2022), and yet others use peptides with a known bioactivity that is different from their target (Charoenkwan *et al.* 2020a, Olsen *et al.* 2020, Agrawal *et al.* 2021).

If we consider the first and second approaches, the model learns to differentiate between peptides with the target bioactivity and random sequences (either protein fragments or peptides). However, the problem is that the model can exploit multiple confounding factors that do not have a direct bearing on the specific bioactivity, but that are related to the differences between generally bioactive peptides and random sequences. In the third approach, the opposite is true, positive and negative peptides may be so similar to each other that the model will be biased towards specific differential features between both bioactivities, hindering its ability to generalize.

In this paper, we explored introducing an intermediate solution: to draw the negative peptides from a database with multiple bioactivities. This approach generates a distribution of negative peptides that is as unbiased as possible (by covering several distinct bioactivities) as to generalize adequately, but that is similar enough to the positive peptide distribution (by also being bioactive peptides) as to minimise confounding factors.

2) **Dataset partitioning**. To evaluate predictive models it is necessary to divide the data into at least three distinct subsets: training, validation, and testing. The independence between the training and testing subsets is essential to obtain a reliable estimation of the future model performance (Walsh *et al.* 2021). To achieve this independence, community guidelines recommend building testing sets that do not share homologous sequences with the training set either by homology reduction (Walsh *et al.* 2021) or homology partitioning (Teufel *et al.* 2023, Fernández-Díaz *et al.* 2024). Despite this, most of the peptide bioactivity predictors reviewed (Rajput *et al.* 2015, Bin *et al.* 2020, Charoenkwan *et al.* 2020a, 2020b, 2021, 2022b, 2022c, Wei *et al.* 2020, Agrawal *et al.* 2021, Pinacho-Castellanos *et al.* 2021, Xiao *et al.* 2021) do not introduce any correction for homology when partitioning their datasets and those that do (Manavalan *et al.* 2019, Olsen *et al.* 2020, Dai *et al.* 2021, Charoenkwan *et al.* 2022a, Chen *et al.*2022, Zhang *et al.* 2022), use high thresholds (80%–90% of sequence identity, Supplementary Table S1), still allowing for similar sequences to be in different sets, which could lead to the overestimation of model performance due to data leakage.

The main difference between homology-based reduction and homology-based partitioning is that in homology-based reduction a first clustering step is performed and only the centroids of the clusters are preserved. In partitioning algorithms, clusters are moved to different partitions so that the sequences within each partition do not have any neighbours in other partitions (e.g. no sequences in the testing set can have a neighbour in the training set). While previous methods (Teufel *et al.* 2023) removed a minimal number of sequences to ensure that partitions are completely independent. We rely on CCPart (Fernández-Díaz *et al.* 2024), a recently developed algorithm that is able to generate independent test sets without sequence removal. With this in mind, we explored the effects of introducing this homology-based dataset partitioning for building testing subsets more suited for evaluating model generalization.

3) **Computational representation of the peptides**. For a predictive model to be able to interpret the peptide sequences, they need to be translated into mathematical objects (vectors or matrices). The reviewed literature offers different options for performing this transformation that include statistics of: Residue composition (Bin *et al.* 2020, Olsen *et al.* 2020, Charoenkwan *et al.* 2021), evolutionary profile (Wei *et al.* 2021), or physico-chemical properties (Bin *et al.* 2020, Charoenkwan *et al.* 2020b, 2022a, Pinacho-Castellanos *et al.* 2021). The consensus that can be drawn from the variety of different descriptor combinations is that each predictive task will require a different set of descriptors. Finding the optimal combination is a crucial and intricate step in the modelling process (Attique *et al.* 2020).

The advent of Protein Language Models (PLMs) like the ESM (evolutionary scale modelling) (Rao *et al.* 2020, Lin *et al.* 2023) or RostLab (ProtBERT, Prot-T5-XL, or ProstT5) (Elnaggar *et al.* 2021, Heinzinger *et al.* 2023) families has allowed for much simpler and richer protein representations. Given a sequence *s*, these models have learned the probability that a residue will appear in position *i* given the rest of the sequence $\left\{s-r_{i}\right\}$, $P(r_{i}|\{s-r_{i}\})$. This probability is related to the concept of conserved and unconserved positions that is often used when analysing multiple sequence alignments (Lin *et al.* 2022). The models are trained on a vast set of sequences from the UniRef (Suzek *et al.* 2007) or BFD (Steinegger *et al.* 2019) databases which include not only protein sequences, but also peptides. Moreover, at least, two prior studies have demonstrated that they can be used for representing peptides outperforming traditional description strategies (Dee 2022, Du *et al.* 2023). However, there are many PLMs varied both in terms of size and learning method and it is not clear which may be the optimal choice for computing peptide representations. In this paper, we continue this line of research by addressing two questions: does model size have an impact on how suitable their representations are for describing peptides? and is there any significant difference between different classes of models?

4) **Model training and hyperparameter optimization**. There are many different algorithms for fitting predictive models to a binary classification task and choosing between them is an extended trial-and-error task. Here, we considered an alternative approach: to use standard tools in the AutoML domain for performing hyperparameter Bayesian optimization of simple machine learning models and ensembling them.

5) **Model evaluation reporting.** The final step in the development of any predictive model is to report how reliable the future predictions of the method are going to be. The main goal of our automated process is to enable researchers without a computational background to leverage the tools, therefore, we structure the output of the pipeline to provide all necessary information for reproducing the training of the model and a summary that offers guidance in how to interpret the different evaluation scores of the model.

These contributions have been integrated into a computational tool and webserver, named AutoPeptideML, that allows any researcher to build their own custom models for any arbitrary peptide bioactivity they are interested in. The webserver requires only minutes to build a predictor and its use is as simple as uploading a dataset with positive examples. It provides an output summary that facilitates the interpretation of the reliability of the predictor generated and it has an additional window supporting the use of the generated models for predicting the bioactivity of any given set of peptides.

## 2 Materials and methods

### 2.1 Data acquisition

Eighteen different peptide bioactivity datasets containing positive and negative samples were used to evaluate the effect of the different methods. These datasets were selected from a previous study, considering the use of the ESM2-8M PLM for general peptide bioactivity prediction (Du *et al.* 2023). The datasets ranged in size from 200 to 20 000 peptides (see Table 1). Here, they are referred to as the ‘original’ datasets.

**Table 1.** Original benchmark datasets.

Dataset	Negative class	Partitioning	Number positives	SOTA Ref.^a^
Antibacterial (Pinacho-Castellanos *et al.* 2021)	Random peptides	Homology maximization	8278	Pinacho-Castellanos *et al.* (2021)
ACE inhibitor (Manavalan *et al.* 2019)	Random protein fragments	Homology reduction (90%)	1299	Manavalan *et al.* (2019)
Anticancer 1 (Agrawal *et al.* 2021)	Antimicrobial peptides	Random	861	Charoenkwan *et al.* (2021)
Anticancer 2 (Agrawal *et al.* 2021)	Random protein fragments	Random	970	Agrawal *et al.* (2021)
Antifungal (Pinacho-Castellanos *et al.* 2021)	Random peptides	Homology maximization	993	Pinacho-Castellanos *et al.* (2021)
Antimalarial 1 (Xiao *et al.* 2021)	Random peptides	Random	139	Charoenkwan *et al.* (2022c)
Antimalarial 2 (Agrawal *et al.* 2021)	Random protein fragments	Random	139	Charoenkwan *et al.* (2022c)
Antimicrobial (Pinacho-Castellanos *et al.* 2021)	Random peptides	Homology maximization	6460	Pinacho-Castellanos *et al.* (2021)
Antioxidant (Olsen *et al.* 2020)	Experimental + random peptides	Homology reduction (90%)	728	Olsen *et al.* (2020)
Antiparasitic (Zhang *et al.* 2022)	Random peptides	Homology reduction (90% for positives and 60% for negatives)	301	Zhang *et al.* (2022)
Antiviral (Pinacho-Castellanos *et al.* 2021)	Random peptides	Homology maximization	2944	Pinacho-Castellanos *et al.* (2021)
Brain–blood barrier crossing (Dai *et al.* 2021)	Random peptides	Homology reduction (90%)	119	Dai *et al.* (2021)
DPPIV inhibitors (Charoenkwan *et al.* 2020a)	Random + Bioactive	Random	665	Charoenkwan *et al.* (2022b)
Anti-MRSA (Charoenkwan *et al.* 2022a)	Random peptides	Homology reduction (80%)	148	Charoenkwan *et al.* (2022a)
Neuropeptide (Bin *et al.* 2020)	Random protein fragments	Homology reduction (90%)	2425	Chen *et al.* (2022)
Quorum sensing (Rajput *et al.* 2015)	Random peptides	Random	220	Wei *et al.* (2020)
Toxic (Wei *et al.* 2021)	Random peptides	Random	1932	Wei *et al.* (2021)
Tumour T-cell antigens (Charoenkwan *et al.* 2020b)	T-cell antigens not associated to disease	Random	592	Charoenkwan *et al.* (2020b)

a SOTA Ref: Reference to best-reported model in the literature.

### 2.2 Dataset with new negative peptides

For each of the original datasets, a new version was constructed using the new definition of negative peptides, termed ‘NegSearch’. The negative peptides were drawn from a curated version of the Peptipedia database ‘APML-Peptipedia’ comprised of 92 092 peptides representing 128 different activities (see Supplementary Fig. S1). To avoid introducing false negative peptides into the negative subset, all bioactivities that may overlap with the bioactivity of interest were excluded (see Supplementary Table S1). To ensure that the negative peptides were drawn from a similar distribution to the positive peptides and thus minimise the number of confounding factors, for each dataset we calculated a histogram of the lengths of its peptides with bin size of 5. Then, for each bin in the histogram, we queried APML-Peptipedia for as many peptides as present in the bin, with lengths between its lower and upper bounds. If there were not enough peptides, the remaining peptides were drawn from the next bin.

### 2.3 Dataset partitioning

Two different partitioning strategies were used to generate the training/testing subsets: (i) random partitioning and (ii) CCPart (Fernández-Díaz *et al.* 2024), a novel homology-based partitioning algorithm which creates an independent testing set ensuring that there are no homologous sequences between training and testing. Briefly, the algorithm calculates pairwise alignments among all dataset sequences to form a pairwise similarity matrix. It then clusters these sequences based on the similarity matrix using the connected components algorithm (Fernández-Díaz *et al.* 2024). Lastly, it iteratively transfers the smallest clusters to the testing set until it reaches the desired size (in our case, 20% of total sequences). This process ensures that there are no sequences in the testing set similar to those in the training set. The datasets generated through this strategy are referred to as ‘NegSearch+HP’.

The CCPart algorithm (Fernández-Díaz *et al.* 2024) achieves two main objectives: (i) it creates a test set completely independent from training, insofar there are no sequences in the test set that are similar (as defined by the threshold) to those in the training set, and (ii) it selects a test set that is as different from the training distribution as possible. This second objective is achieved by selecting the smallest clusters. The advantage of this decision is that because the clusters selected are small, we can fit more of them into the test set improving its diversity. On the other hand, the smaller a cluster is, the fewer neighbours it has and, consequently, the more unique the sequence is within the dataset. Overall, the algorithm attempts to simulate the real world scenario where the model is used to predict sequences different from those in the training set.

In both cases, (i) randomly partitioned or (ii) homology partitioned, the training set is further subdivided into 10 folds for cross-validation. This second division relies on random stratified partitioning, to create 10 cross-validation folds.

### 2.4 Pairwise sequence alignments

The pairwise sequence alignments were calculated using the MMSeqs2 software with prior k-mer prefiltering (Steinegger and Söding 2017). We considered that two peptides were similar if they had a sequence identity above 30% using the longest sequence as denominator.

### 2.5 Peptide representations

In order to evaluate the PLM peptide representations, the following methods (Rao *et al.* 2020, Elnaggar *et al.* 2021, Heinzinger *et al.* 2023, Lin *et al.* 2023) were evaluated: ESM2-8M, ESM2-35M, ESM2-150M, ESM2-650M, ESM1b, ProtBERT, Prot-T5-XL-UniRef50, ProstT5 (sequence mode), and one-hot encoding as a non-PLM-based baseline.

PLMs generate as output a matrix *M* with shape n×e, where *n* is the number of residues in the peptide and *e* is the model embedding size (in this study e∈[320,1280], depending on the model). Each row in this matrix corresponds to a residue-level representation. We obtain a peptide-level representation *r* by averaging across all residues: $r=\frac{1}{n}\sum_{i=1}^{n}M_{i}$ (Dee 2022, Du *et al.* 2023). Please note that *r* is a vector with *e* dimensions.

### 2.6 Model training and hyperparameter optimization

In order to evaluate the model training and hyperparameter optimization step, hyperparameter optimization through bayesian optimization (Akiba *et al.* 2019) was performed separately for K-nearest neighbours (KNN), light gradient boosting machine (LightGBM) and random forest classifier (RFC) and all models were ensembled (see Supplementary Table S2 for more details about the hyperparameter optimization). The optimization aims to maximize model performance [measured as the average Matthew’s correlation coefficient (Chicco *et al.* 2021) across the 10 cross-validation folds]. The optimization was conducted separately for each of the three models, leading to one optimal hyperparameter configuration per algorithm (three in total). After hyperparameter optimization, each of the three models was trained against each of the 10 cross-validation folds using the optimal configuration. Thus, the final ensemble contained 10 instances (one per cross-validation fold) of each of the three models for a total of 3×10=30 models.

Final ensemble predictions were the average of all 30 individual predictions. This strategy is referred to as ‘Optimized ML Ensemble’ or OMLE throughout the text. The three learning algorithms we used were chosen to provide a diverse representation of simple machine learning algorithms with computationally efficient implementations. We decided to use an ensemble, because it has been shown that for small datasets it leads to more robust predictors (Dvornik *et al.* 2019).

Our system was compared against an amended version of the UniDL4BioPep (Du *et al.* 2023) framework, which we named ‘UniDL4BioPep-A’ [more details about the architecture of the model can be found in the original publication (Du *et al.* 2023) and are summarized in Supplementary Table S3]. This amendment differs from the original in that, following community guidelines (Walsh *et al.* 2021), it used 10-fold cross-validation to determine the best possible checkpoint, instead of the hold-out testing set.

Every training experiment was run three times in order to get a crude estimation of the variability between experiment replications. The number of replicates is too small for proper statistical significance comparison, but the experimental design was constrained by the computational cost of each individual experiment run.

### 2.7 Model evaluation metrics

Model performance was measured in terms of Matthew’s correlation coefficient (MCC), which is a binary classification metric that is specially recommended for measuring model performance in datasets with imbalanced labels (different number of positive and negative samples) (Chicco *et al.* 2021, Chicco and Jurman 2023). Most datasets considered in the study have a balanced number of positive and negative labels. Therefore, for the purposes of model evaluation, we have defined any prediction with a probability score >0.5 as positive and lower or equal as negative.

### 2.8 Calculation of peptide physico-chemical properties

To better describe the composition of the datasets used throughout the study we calculated the distribution of different physico-chemical properties of the peptides. The properties considered were: The aliphatic index using the method described by (Ikai 1980); the Boman potential interaction index using the method described by (Boman 2003); the charge and isolectric point using the methods described by (Sillero and Maldonado 2006); the hydrophobic moment using the method described by (Eisenberg *et al.* 1984); and the predicted structural class using the method described by (Zhou and Assa-Munt 2001). The calculations of all aforementioned algorithms were performed using the corresponding implementations available in (Larralde n.d.) with default settings.

## 3 Results and discussion

We have focused our study of peptide bioactivity prediction in the binary classification task of discriminating between peptides that show a specific biologically relevant property or function and those which do not. There are three reasons informing this choice: (i) there are more datasets available for binary classification than regression, making the benchmarking more comprehensive, (ii) the intepretation of regression metrics like root mean squared error (RMSE) is less intuitive than metrics for binary classification in balanced datasets, and therefore less suitable for the target non-expert audience, and (iii) multi-class or multi-label problems can be formalized as sets of binary classification problems (García-Pedrajas and Ortiz-Boyer 2011).

### 3.1 Effect of the sampling strategy for gathering negative peptides

We started by examining the effect of the new sampling strategy for gathering negative peptides.

#### 3.1.1 The new negative peptides have a distribution of physico-chemical properties more similar to that of the positives

We have first examined the hypothesis that sampling negative peptides from the APML-Peptipedia database of bioactive peptides would lead to negative peptides with distributions more similar to those of the positive peptides. We calculated the distributions for different physico-chemical properties of the datasets and compared the positives with the original negatives and the new negatives (see Supplementary Figs S2–S17).

The results show that, generally, the distribution of the new negatives is closer to the distribution of the positive peptides, specifically in the cases of the Antibacterial, ACE inhibitor, Antifungal, Antimalarial, Antimicrobial, Antioxidant, Antipara-sitic, Brain-blood barrier crossing, DPPIV inhibitor, and Toxicity. In the rest of the datasets, the distribution resembles much more closely that of the original negatives.

#### 3.1.2 The introduction of the new negative peptides leads to more challenging modelling problems

**Figure 2.**
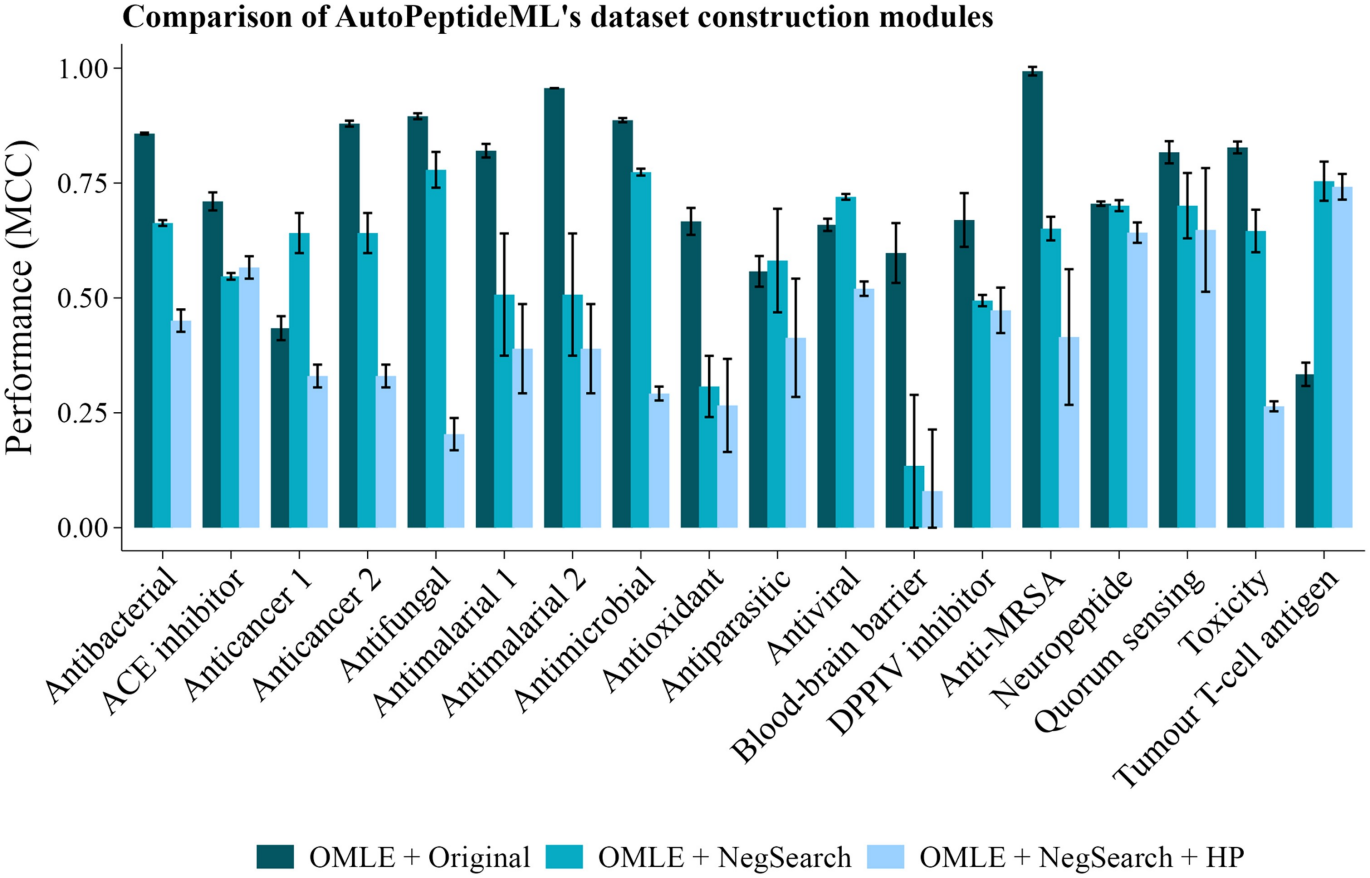
Evaluation of AutoPeptideML’s dataset construction modules. Error bars reflect the standard deviation across three replicates. OMLE: optimized ML ensemble; Original: original benchmark; NegSearch: dataset with new negative peptides; HP: homology-based dataset partitioning module.

We then examined the effect that the introduction of the new negative peptides had in the complexity of the modelling problem. With everything else being identical, Fig. 2 clearly shows how the introduction of the new negative peptides leads to a more challenging modelling problem, this is most likely due to the reduction of confounding factors that the model can exploit to discriminate between the positive and negative classes.

It is particularly interesting to consider the change in the dataset pairs Anticancer 1 and 2, and Antimalarial 1 and 2 as the original datasets were built from the same sets of positive peptides, but relied on different sampling strategies for obtaining the negative peptides. Briefly, Anticancer 1 drew its negatives from a database of antimicrobial peptides [which are known to overlap with the anticancer peptides (Tornesello *et al.* 2020)] and Anticancer 2 drew them from a database of random protein fragments. This is reflected in Fig. 2 where Anticancer 1—Original demonstrates lower performance than Anticancer 2—Original. It is noteworthy that the NegSearch dataset achieves an intermediate performance between them, as was to be expected from the NegSearch negative sampling being an intermediate definition between a specific bioactivity and random peptide sequences. Similarly, Antimalarial 1—Original draws its negative peptides from a collection of random peptides, while Antimalarial 2—Original draws them from a collection of protein fragments. Figure 2 shows how the more restrictive that the negative peptide sampling is the lower the model apparent performance, with Antimalarial 2—Original achieving the highest apparent performance and Antimalarial—NegSearch the lowest.

Overall, the results indicate that the choice of sampling method for acquiring the negative peptides has an important effect on the perceived model performance. In the end, the optimal sampling method will depend in the intended use for the model and whether it will be applied to protein fragments, random peptides or to distinguish between different peptide bioactivities. However, our experiments suggest that sampling from a collection with peptides with diverse bioactivities offers a balance between specificity and future generalization, particularly, when the future target distribution is unknown at time of model development.

### 3.2 Effect of homology-based partitioning

We next examined the effect of introducing the homology-based partitioning algorithm for generating the training and test sets.

#### 3.2.1 The training and test sets are not independent in the original datasets

We started by analysing the interdependence between training and test sets in the original datasets. We measured interdependence as the proportion of peptides in the training set with at least one similar peptide in the test set. We classified two peptides as similar if they have >30% sequence identity in the pairwise local alignment. The results compiled in Table 2 indicate that for 13 of the 18 original datasets, at least 10% of the peptides in the training set are similar to sequences in the testing set, compromising their independence. If we consider the datasets (see Table 1) for which homology-based correction was used (ACE inhibitor, Antioxidant, Antiparasitic, Anti-MRSA, and Neuropeptide), we observe that only two of them have <10% interdependence, which highlights the need for introducing similarity correction techniques at low thresholds.

**Table 2.** Training-testing interdependence analysis of all datasets.

Dataset	**Original** (%)^a^	**NS** (%)^a^	**NS+HP** (%)^a^
Antibacterial	36	64	0
ACE inhibitor	1	3	0
Anticancer 1	59	50	0
Anticancer 2^b^	30	∼	∼
Antifungal	34	58	0
Antimalarial 1	41	15	0
Antimalarial 2^c^	10	∼	∼
Antimicrobial	47	63	0
Antioxidant	1	0	0
Antiparasitic	11	32	0
Antiviral	26	44	0
Blood-brain barrier	4	4	0
DPPIV inhibitor	5	4	0
Anti-MRSA	15	34	0
Neuropeptide	24	24	0
Quorum sensing	18	9	0
Toxicity	56	56	0
Tumour T-cell antigens	0	3	0

a The percentages correspond to the proportion of training sequences with at least one similar sequence (sequence identity >30%) in the testing set. Columns correspond to the different datasets constructed: Original datasets, the NegSearch datasets (NS) and the NegSearch datasets with homology-based partitioning (NS+HP).

b Equivalent to Anticancer 1 for NS and NS+HP.

c Equivalent to Antimalarial 1 for NS and NS+HP.

The introduction of the new negatives does not reduce the interdependence between training and test sets, but it can even increase in some cases. The biggest increments are observed in the Antibacterial, Antifungal, Antimicrobial, Antiviral, and Antiviral datasets. These datasets all have been partitioned using a homology maximization algorithm (see Table 1) that creates a test set with representatives from all clusters in training, this representatives are evenly sampled and thus the interdependence between the two subsets while not reduced it is bounded. In the ‘NS’ datasets, training and test sets are randomly partitioned, and therefore members from highly connected clusters can be overrepresented in the test set, thus leading to the observed increase in the interdependence.

#### 3.2.2 Independent test sets lead to more challenging evaluation

Table 2 shows how that the training and test sets are completely independent from each other when the CCPart algorithm is used for homology-based dataset partitioning.

Figure 2 clearly shows how the independent test sets lead to a much more challenging evaluation with a significant drop in model performance in most datasets. This result suggests that previous studies have tended to overestimate the performance of the models when applied to real-world sequences different from those present in their training set.

In this study, we have considered as similarity metric the sequence identity in local pairwise alignments when performing the homology-based partitioning, following the prior studies that introduced any type of homology-based correction technique referenced in Table 1, which all use sequence identity. The results obtained showcase the importance of accounting for the similarity between training and test partitions to properly evaluate model out-of-distribution generalization. Peptides being entities halfway between proteins and small molecules, also support other similarity metrics based on their physico-chemical properties or chemical structure that may be more suitable for partitioning (Fernández-Díaz *et al.* 2024, Orsi and Reymond 2024). We keep the exploration of alternative similarity metrics for peptide dataset partitioning for future work.

#### 3.2.3 Homology-based partitioning generates sufficiently diverse test sets

One possible problem with homology-based partitioning is that it may lead to the creation of test sets with very few similarity clusters, thus compromising evaluation. A comparison of the number of clusters present in the training and test set before and after the introduction of new negatives and homology-based partitioning (see Supplementary Table S4) shows that though the number of clusters in the test set always diminishes, in most cases the number of clusters in the test set still represents approximately 20% of the number of clusters in training, and it never represents <10%. This confirms that the CCPart algorithm is able to generate sufficiently diverse test sets.

### 3.3 Protein language models as peptide representation methods

Recent studies have reported the use of PLMs for predicting peptide bioactivity (Dee 2022, Du *et al.* 2023), however, they have not been compared to a naive baseline representation like one-hot encoding; nor has there been an evaluation on which PLM may be more suited for peptide representation. All experiments are conducted with the NegSearch+HP datasets to ensure that we were properly evaluating model generalization.

#### 3.3.1 Baseline

**Figure 3.**
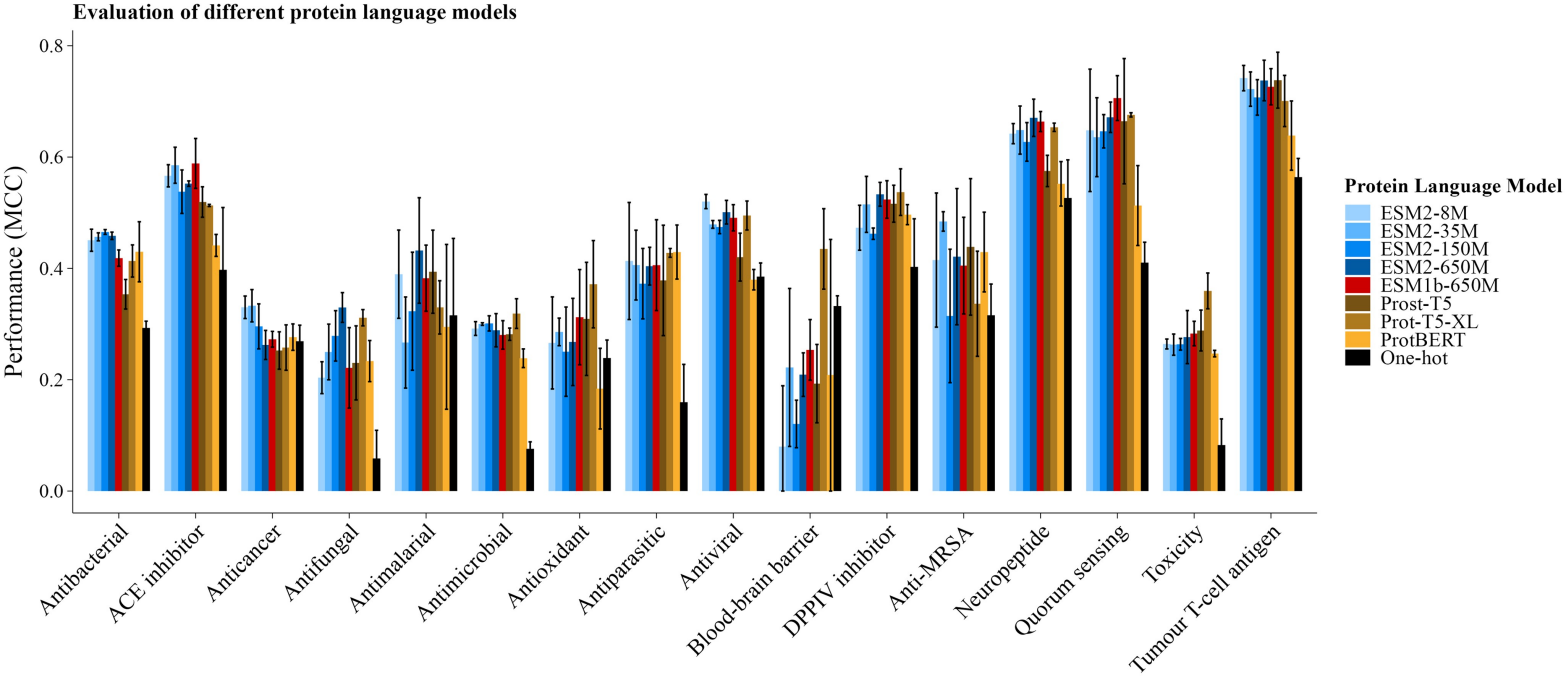
Evaluation of different protein language models. Error bars reflect the standard deviation across three replicates.

First, we compare the PLMs to a naive baseline representation (one-hot encoding). Figure 3 shows that generally PLMs are significantly better representation methods across datasets, though in specific cases one-hot encoding appears to achieve similar performance. There are different idiosyncrasies within those datasets that may explain the behaviour, e.g. in the Blood-brain barrier experiments the number of training peptides is really small (∼200, see Table 1) which leads to a lot of instability between the different runs (as can be seen by the size of the error bars).

#### 3.3.2 Model size

We evaluated four different PLM models from the ESM family with increasing size: ESM2-8M (8 million parameters), ESM2-35M (35 million parameters), ESM2-150M (150 million parameters), ESM2-650M (650 million parameters). We also evaluated ESM1b-650M (650 million parameters), from a previous version of ESM. Figure 3 shows that there is no significant difference between models across all datasets and no correlation between model size and performance.

This observation appears contrary to the established consensus that bigger PLMs tend to perform better (Rao *et al.* 2019, Elnaggar *et al.* 2021, Lin *et al.* 2023). It is important to note that those studies focused on a very particular use of the PLMs known as full-model transfer learning (Li *et al.* 2024). However, in our experiments, we relied on representation transfer instead (Unsal *et al.* 2022, Li *et al.* 2024). The main difference between both regimes is that in full-model transfer learning every parameter in the model is adjusted (fine-tuned) for the downstream task, whereas in representation transfer, the internal parameters of the model do not change. There are two main consequences that derive from this distinction.

First, full-model fine-tuning tuning requires the model to run several times through the training data to iteratively optimize its internal parameters. This is a computationally intensive operation, and the cost increases with model size. Representation transfer, in contrast, only requires a single run through the training data to compute the representations and is thus much faster and does not require specialized hardware like GPUs. Furthermore, when working with small datasets sizes (like most peptide datasets), there is less risk of overfitting with representation transfer (only the parameters of the downstream model are optimized) than with full-model fine-tuning (where both the model parameters, $8-650\times 10^{6}$, and the downstream models are optimized). We decided to focus on representation transfer for this study because of these two reasons.

Second, the more parameters a model has, the greater its learning capacity will be. This learning capacity can only be accessed in full-model fine-tuning as it allows for the optimization of the internal parameters of the model. Thus, observing no correlation between model size and downstream performance in a representation transfer setting is not necessarily inconsistent with the prior literature. The question of whether the established PLM scaling rules for full-model transfer learning when modelling peptide sequences remains unanswered and is left for future work.

#### 3.3.3 Type of model

We further compared the ESM models to the main models from the RostLab family: ProtBERT, Prot-T5-XL-UniRef50, and Prost-T5. Figure 3 shows that even though for certain datasets there might be significantly better models, when the effect is analysed across all datasets there is no significant difference between the different models or families.

All things considered, the ESM2-8M model achieves a commendable balance between enhanced performance relative to one-hot encoding and minimal computational requirements. In any case, the optimal performance will likely be achieved when traditional representation methods are combined with PLM representations. A systematic study on the optimal way to combine these types of features is kept for future work.

### 3.4 Optimized machine learning ensemble and PLM representations as an alternative to highly engineered approaches

We compared the performance of two general purpose frameworks: a deep learning-based model [UniDL4BioPep-A (Du *et al.* 2023) and Supplementary Table S3] and our optimized machine learning ensemble (OMLE).

#### 3.4.1 Comparison to handcrafted models

**Figure 4.**
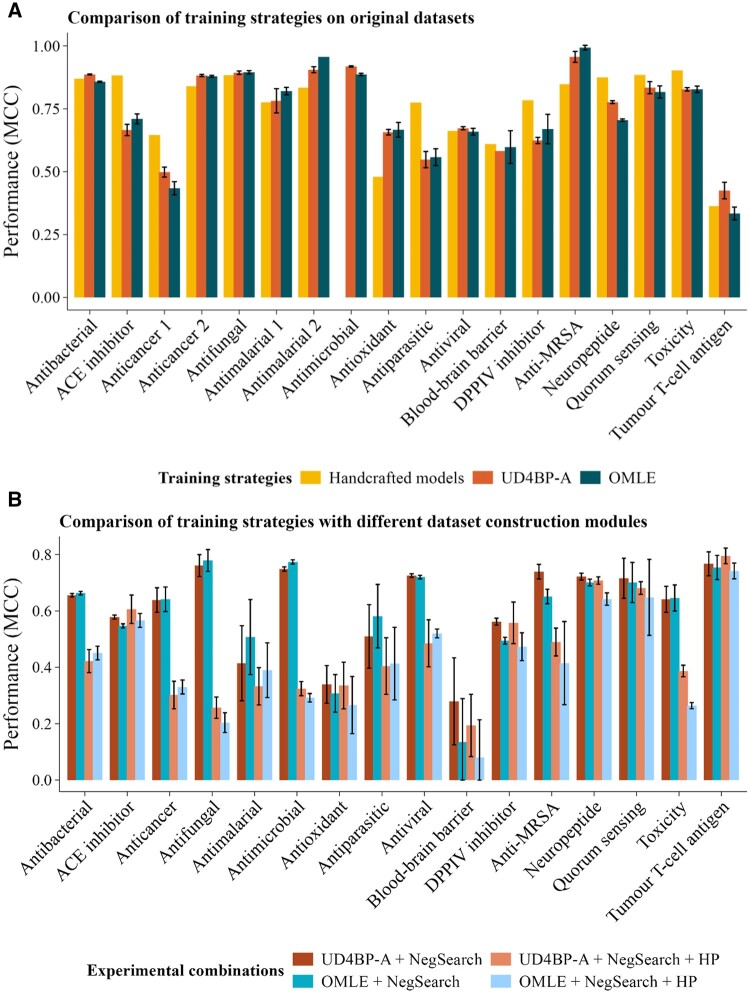
(A) Comparison of training strategies on original datasets. (B) Comparison of training strategies with different dataset construction modules. Error bars reflect the standard deviation across three replicates. OMLE: optimized ML ensemble; UD4BP-A: UniDL4BioPep-A.

Figure 4A (see Supplementary Table S8 for alternative metrics) shows that when applied to a literature-derived benchmark set of datasets, the two general purpose PLM-enabled bioactivity predictors have a performance comparable with the self-reported performance of the best handcrafted models for each specific dataset (see Table 1 for the reference of each of the models). Moreover, our proposed AutoML solution (OMLE) was able to out-perform the handcrafted models on 6 out of 17 benchmark datasets for which data was available and was not significantly different for another 2.

These results show that the combination of the PLM representations and the OMLE learning strategy, provide a fast, convenient, and competitive alternative to highly engineered approaches with handcrafted models and peptide representations that require both domain and technical expertise.

In any case, the results obtained with our approach, though solid with the out-of-the-box configuration, can be further improved by combining the PLM representations with traditional representations and defining OMLEs with wider or narrower sets of learning algorithms and hyperparameter spaces.

#### 3.4.2 Comparison of an optimized ML ensemble with a neural network

When compared with Fig. 4A, Fig. 4B (see Supplementary Table S9) shows that when the new sampling strategy for gathering negative peptides is introduced, both ML and neural network general purpose models show an equivalent drop in apparent performance, reflecting the more challenging task of predicting a specific bioactivity against peptides from a diverse collection of bioactivities. The performance drops further in both models when homology-based partitioning is introduced. Remarkably, there is no significant evidence of greater overfitting on the part of the DL model, despite the small dataset sizes, this might be due to the relatively small size of UniDL4BioPep-A. Overall, these results allow us to conclude that OMLE achieves comparable performance to a more complex neural network model, while being both more user-friendly and computationally efficient.

### 3.5 AutoPeptideML

**Figure 5.**
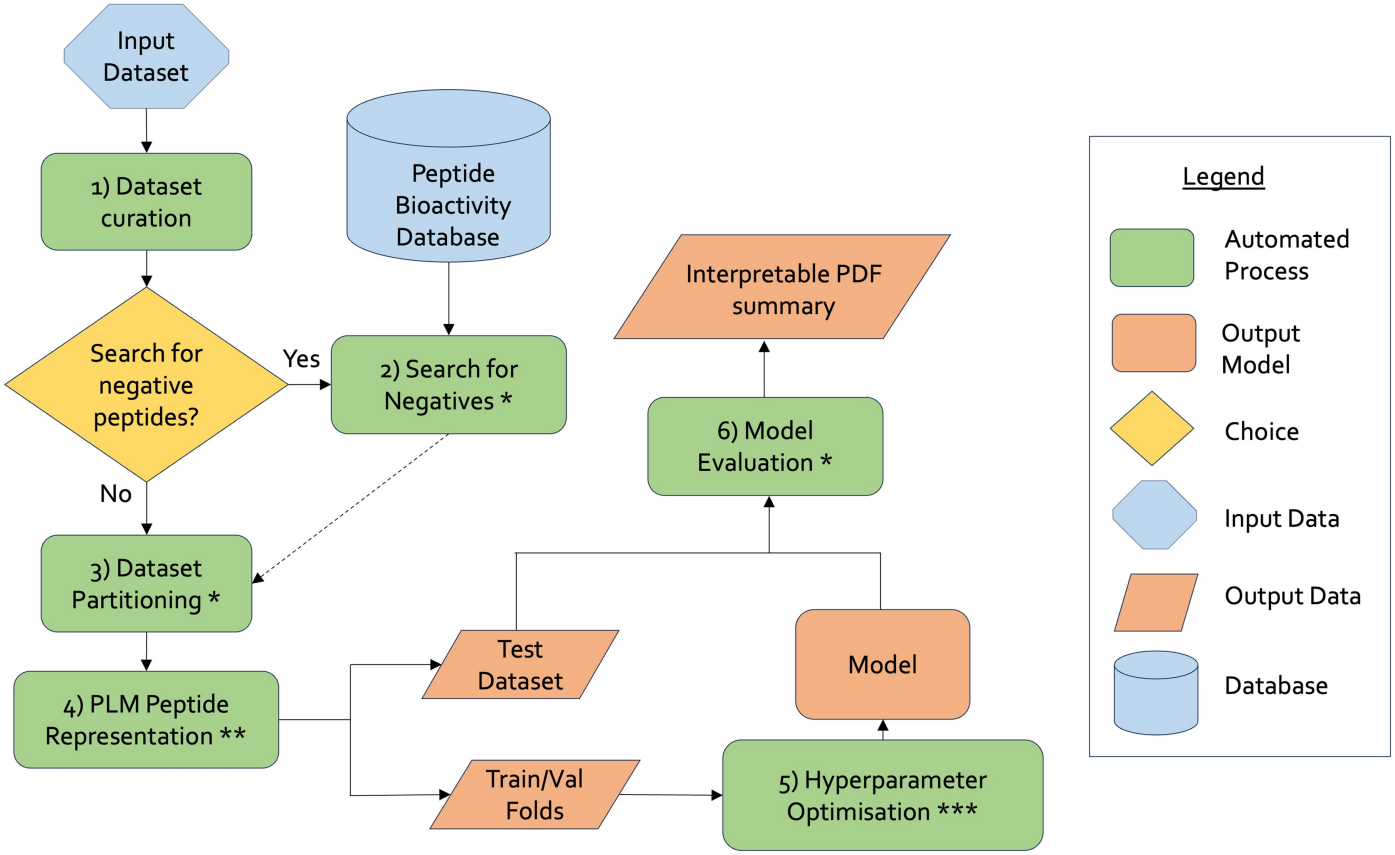
Visual summary of the AutoPeptideML workflow. *: Steps based on the results showcased in Fig. 2. **: Step based on the results showcased in Fig. 3. ***: Step based on the results showcased in Fig. 4.

All the findings described thus far, were used to guide the development of AutoPeptideML, a computational tool and webserver that allows researchers to easily build strong peptide bioactivity predictors and provide a robust evaluation that complies with community guidelines. Figure 5 provides an overview of the final AutoPeptideML workflow.

The primary objective behind the design of AutoPeptideML is to provide a user-friendly tool that does not require extensive technical knowledge to use, while still remaining highly versatile. This is achieved through a pipeline that guarantees compliance with community guidelines such as DOME (Data, Optimization, Model, and Evaluation) (Walsh *et al.* 2021), ensuring a robust scientific validation (see Supplementary S14).

Users are free to define the number of models that should be included in the hyperparameter optimization, as well as their hyperparameter search space. AutoPeptideML supports the following algorithms: K-nearest neighbours (KNN), light gradient boosting (LightGBM), support vector machine (SVM), random forest classification (RFC), extreme gradient boosting (XGBoost), simple neural networks like the multi-layer perceptron (MLP), and 1D-convolutional neural networks (1D-CNN). Model selection and HPO are conducted simultaneously in a cross-validation regime so that the metric to optimize is the average across *n* folds. Thus, the system is never exposed to the testing set, which is kept unseen until the final model evaluation (Walsh *et al.* 2021). The system also supports all PLM models used throughout the study.

AutoPeptideML can be used in two regimes: *Model builder* and *Prediction*. In the first mode new predictive models are created automatically from a single file with known positive peptides for the bioactivity of interest. In the second mode, any predictive model generated through the *model builder* can then be used to predict for each peptide in a dataset the likelihood that it possesses the desired bioactivity.

The outputs that the program generates are:

- *Model builder:* When used to develop new predictors, AutoPeptideML outputs a model fitted to predict the bioactivity of interest, a folder with all information necessary for reproducing the model, and an interpretable summary of the model capabilities see Supplementary S14.
- *Prediction:* AutoPeptideML can also be used to leverage existing predictors. In this case, it outputs a list of the problem peptides sorted in descending order of predicted bioactivity (higher bioactivity first) and a measure of the uncertainty of each prediction.

AutoPeptideML is a tool that enables teams of experimental researchers without access to modelling expertise to quickly and easily build and interpret custom models to integrate into their experimental workflows. It can also be helpful for computational researchers to generate quick and robust baselines at the early stages of a new modelling project against which to compare any new methods. Moreover, the AutoPeptideML Python API allows for using any combination of representations that the researcher may desire to include (both traditional and PLM-based). Thus, it can assist both domain experts and computational scientists by providing a flexible and easy-to-use end-to-end modelling pipeline, allowing the former to easily construct new models and the latter to quickly run experiments and compare between different representation methods or modelling algorithms within a robust and reproducible environment.

## 4 Conclusions

The definition of the negative class used for building peptide bioactivity predictors has a significant impact on the model performance of up to 40% and has to be controlled in order to properly interpret model predictions. Here, we introduced a negative sampling strategy that gathers negative peptides from a collection of peptides with diverse bioactivities which has been shown to achieve a balance between the strengths and weaknesses of current methods in terms of the specificity and interpretability of the predictions, and the reliability of the estimation of future model performance.

The partitioning strategy used to generate training and test subsets impacts the evaluation of model generalization significantly and the introduction of homology-based partitioning algorithms can lead to a drop in perceived model performance of up to 50% when compared to random partitioning. The magnitude of these effects suggests that the model performance has been overestimated in most previous studies.

Using protein language models (PLMs) for computing peptide representations is a significantly better strategy than using a one-hot encoding (a naive representation) for most of the datasets considered. This underscores the potential of PLMs to compute peptide representations, in line with previous studies. Surprisingly, there is no significant correlation between model size and the performance observed, nor among different models. This marks a first step towards understanding the limitations of PLM scaling rules as it pertains their use for modelling peptide sequences.

The combination of PLM peptide representations and an optimized ensemble of simple ML models reaches state-of-the-art performance when compared both to an alternative general-purpose-framework and dataset-specific, handcrafted models across a set of 18 different datasets. Furthermore, there is no significant difference between using an ensemble of simple ML algorithms and more complex DL algorithms (UniDL4BioPep-A), even though the former is more computationally efficient.

We present AutoPeptideML as a computational tool and webserver that allows researchers without technical expertise to develop predictive models for any custom peptide bioactivity. It also facilitates compliance with community guidelines for predictive modelling in the life-sciences. It is able to handle several key steps in the peptide bioactivity predictor development life-cycle including: (i) data gathering, (ii) homology-based dataset partitioning, (iii) model selection and hyperparameter optimization, (iv) robust evaluation, and (v) prediction of new samples. Further, the output is generated in the form of a PDF summary easily interpretable by researchers not specialized in ML; alongside a directory that ensures reproducibility by containing all necessary information for re-using and re-training the models. All data and code are made available to enable the reproducibility of the results in this work.

The foundational principles underlying the issues described and solutions implemented throughout this study are relevant for the application of trustworthy ML predictors for any other biosequence (e.g. DNA, RNA, proteins, peptides, DNA methylation, etc.) and their automation facilitates the rigorous evaluation and development of new models by researchers not specialized in ML.

## Supplementary Material

btae555_Supplementary_Data
