# Supplementary material for: AutoPeptideML: a study on how to build more trustworthy peptide bioactivity predictors
Source: Bioinformatics. 2024 Sep 18;40(9):btae555. doi: 10.1093/bioinformatics/btae555 (PMC11438549; doi:10.1093/bioinformatics/btae555)
Supplement: btae555_Supplementary_Data [file btae555_supplementary_data.zip › summary.pdf]

AutoPeptideML output summary

1. Introduction

This document is automatically generated from each run of the AutoPeptideML software and it is meant to provide an easy guide into the interpretation of the results obtained. General qualitative comments (e.g., "the MCC score obtained is between 0.25-0.5, which indicates a low correlation and model predictions will not be greatly reliable") are given as a common-sense guideline and the actual criteria for considering a model acceptable may depend on the target application and current state-of-the-art.

2. Confusion matrix and main performance metrics

2.1. Confusion matrix

The confusion matrix is the simplest way to visualize the behaviour of the model. The rows describe the true labels of the samples, which can be Positive or Negative; the columns describe the predicted labels from the ensemble.

- **First quadrant (upper-left corner):** describes the True Negative predictions (TN) that is to say samples that are negative which are predicted as negative by the model.
- **Second quadrant (upper-right corner):** describes the False Positive predictions (FP), which are negative samples that are erroneously predicted as positives. If this error is high usually indicates an over-sensitive predictor.
- **Third quadrant (lower-left corner):** describes the False Negative predictions (FN), which are positive samples that are erroneously predicted as negatives. If this error is high usually indicates a highly specific predictor.
- **Fourth quadrant (lower-right corner):** describes the True Positive predictions, which are positive samples predicted as positives.

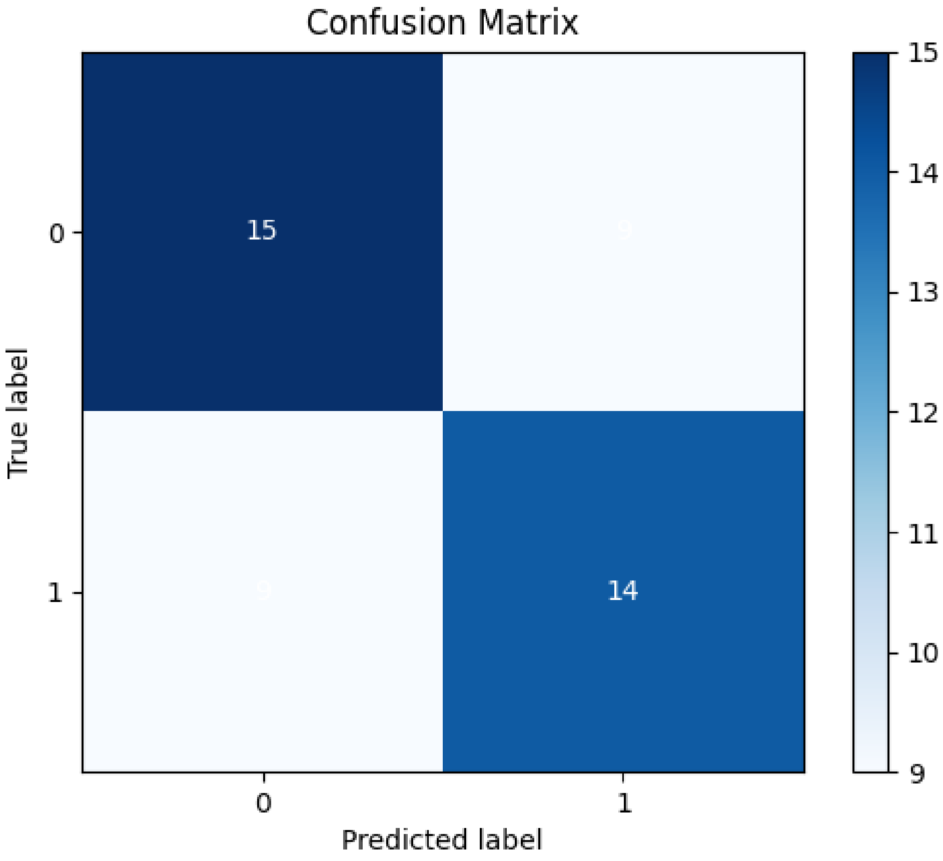

Confusion matrix

2.2. Model performance metrics

The confusion matrix can be analysed in different ways depending on what properties of the predictor we are most interested in. The following list contains the main performance metrics used when describing ML predictors, a formula relating them to the confusion matrix above, and a explanation of what they tell us about the model.

- **Accuracy:**
    - *What is it?* proportion of correct predictions among all predictions.
    - *How is it calculated?* `(TP+TN)/(TP+TN+FP+FN)`
    - *What does it say about the model?* How often is it right.
    - *When to use?* Only when working with a balanced dataset (same number of positive and negative samples, default AutoPeptideML run with search for bioactive negatives and homology partitioning). If dataset is not balanced check `evaluation_data/test_scores.csv` for `balanced_accuracy` which is a variation that takes into account the imbalance between the labels.
    - *Value:* `0.617`
    - *Interpretation of value:*
        - Worse than random: `0-0.45`
        - Random model: `0.45-0.55`

        - Bad model: `0.55-0.7`
        - Acceptable model: `0.7-0.8`
        - Good model: `0.8-0.9`
        - Really good model: `0.9-0.97`
        - Too good a model (please make sure training and evaluation sets are independent): `>0.97`

- **Sensitivity or recall:**

    - *What is it?* proportion of positive samples predicted as positive among all positive samples.
    - *How is it calculated?* `(TP)/(TP+FN)`
    - *What does it say about the model?* How likely it is to misclassify a positive sample as negative. May be relevant when the consequence of missing positives is important (e.g., a cancer diagnostics tool).
    - *When to use?* Only when working with a balanced dataset (same number of positive and negative samples, default AutoPeptideML run with search for bioactive negatives and homology partitioning). If dataset is not balanced check `evaluation_data/test_scores.csv` for `recall_weighted` which is a variation that takes into account the imbalance between the labels.
    - *Value:* `0.609`
    - *Interpretation of value:*

        - Worse than random: `0-0.45`
        - Random model: `0.45-0.55`
        - Bad model: `0.55-0.7`
        - Acceptable model: `0.7-0.8`
        - Good model: `0.8-0.9`
        - Really good model (check that specificity is, at least, good): `0.9-0.97`
        - Too good a model (please make sure training and evaluation sets are independent, also check that specificity is, at least, good): `>0.97`

- **Specificity or precision:**

    - *What is it?* proportion of positive predictions that were actually true.
    - *How is it calculated?* `(TP)/(TP+FP)`
    - *What does it say about the model?* How likely it is to misclassify a positive sample as negative. May be relevant when the aim is to reduce the number of samples to further analyse (e.g., when conducting virtual screening on large databases).
    - *When to use?* Only when working with a balanced dataset (same number of positive and negative samples, default AutoPeptideML run with search for bioactive negatives and homology partitioning). If dataset is not balanced check `evaluation_data/test_scores.csv` for `precision_weighted` which is a variation that takes into account the imbalance between the labels.
    - *Value:* `0.609`
    - *Interpretation of value:*

        - Worse than random: `0-0.45`
        - Random model: `0.45-0.55`
        - Bad model: `0.55-0.7`
        - Acceptable model: `0.7-0.8`
        - Good model: `0.8-0.9`
        - Really good model (check that sensitivity is, at least, good): `0.9-0.97`
        - Too good a model (please make sure training and evaluation sets are

independent, also check that sensitivity is, at least, good): `>0.97`

- **F1:**
    - *What is it?* harmonic mean between sensitivity and precision.
    - *How is it calculated?* `(1/2) * (TP)/[2*TP + (FP+FN)]`
    - *What does it say about the model?* Overall model performance, conceptually similar to accuracy.
    - *When to use?* Only when working with a balanced dataset (same number of positive and negative samples, default AutoPeptideML run with search for bioactive negatives and homology partitioning). If dataset is not balanced check `evaluation_data/test_scores.csv` for `f1_weighted` which is a variation that takes into account the imbalance between the labels.
    - *Value:* `0.609`
    - *Interpretation of value:*
        - Worse than random: `0-0.45`
        - Random model: `0.45-0.55`
        - Bad model: `0.55-0.7`
        - Acceptable model: `0.7-0.8`
        - Good model: `0.8-0.9`
        - Really good model: `0.9-0.97`
        - Too good a model (please make sure training and evaluation sets are independent): `>0.97`

- **Matthew's correlation coefficient:**
    - *What is it?* correlation between the predictions of the model and the actual true labels.
    - *How is it calculated?* `(TP*TN · FP*FN) / ·(TP+FP)(TP+FN)(TN+FP)(TN+FN)`
    - *What does it say about the model?* Overall model performance, conceptually similar to accuracy.
    - *When to use?* Any case, particularly with binary classification.
    - *Value:* `0.234`
    - *Interpretation of value:*
        - Worse than random: `< -0.2`
        - Random model: `-0.2-0.2`
        - Bad model: `0.2-0.3`
        - Acceptable model: `0.3-0.4`
        - Good model: `0.4-0.7`
        - Really good model: `0.7-0.95`
        - Too good a model (please make sure training and evaluation sets are independent): `>0.95`

3. Calibration curve

The calibration curve indicates whether the `score` obtained from the predictions of the ensemble can be considered as a probability of the sample being positive, i.e., whether a higher `score` represents a greater likelihood for the sample to be positive.

If the `Classifier 1` curve follows the doted diagonal curve (`Perfectly calibrated`) then the `score` values can be considered as a probability. Otherwise, they cannot. If the curve approximates the diagonal in a region

and not in other (e.g., below 0.5 is well calibrated and above 0.5 it is not), it can only be considered as a probability if the `score` falls within that region.

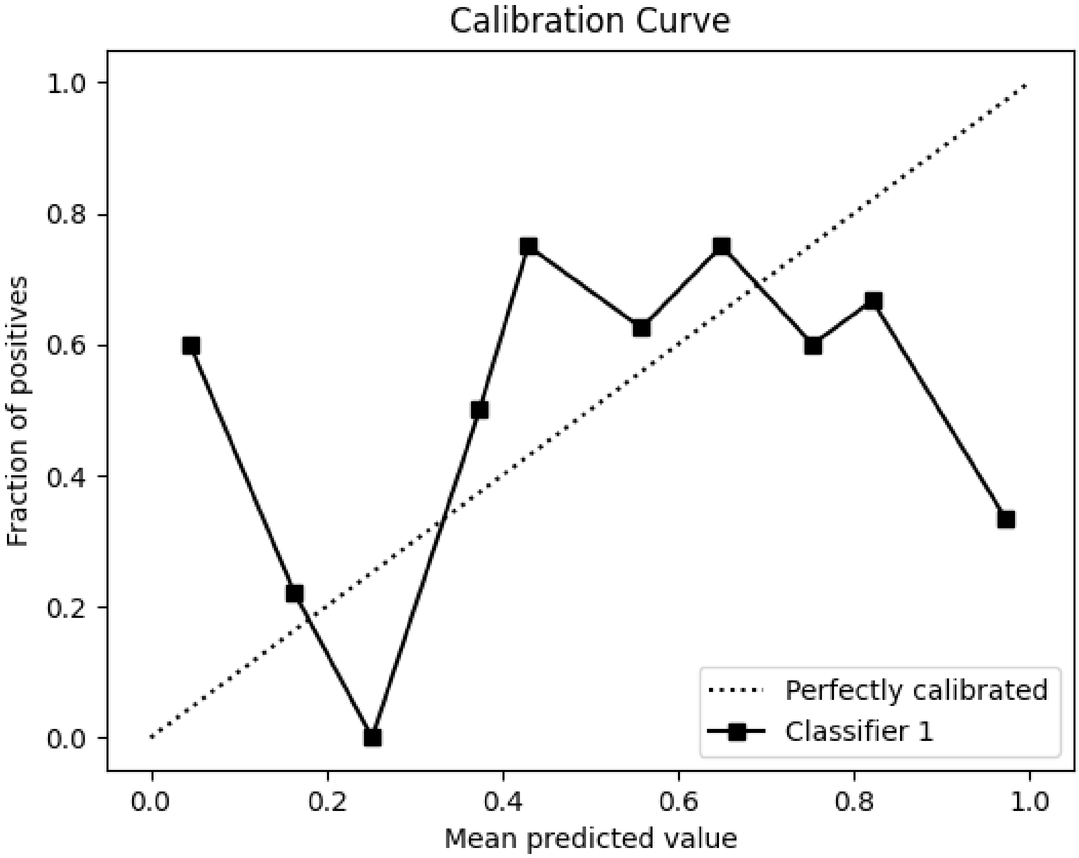

Calibration curve

4. Receiver-operating characteristic curve (ROC)

The ROC curve represents the positive sensitivity (see above **sensitivity or recall**) of the predictor against the false positive rate (the proportion of observations that are incorrectly predicted to be positive out of all negative observations: `FP/(TN+FP)`). The closer that the curve is to the upper-left corner the better the model is. There is also a metric associated to this curve the **AUROC** (area under the ROC) which is often used in the ML community.

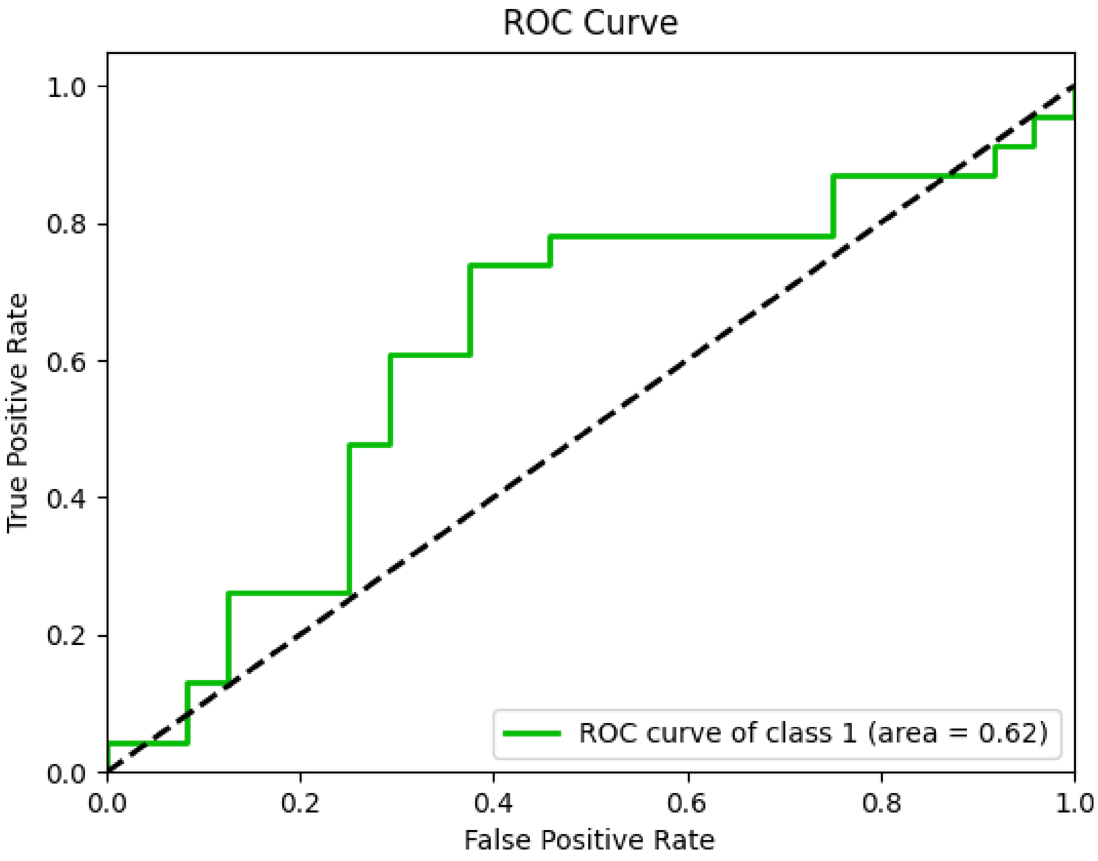

AUROC

5. Precision-recall curve

The precision-recall represents the specificity/precision against the sensitivity/recall and provides an idea of the trade-offs existing in the model between both measurements. The area under the curve is also a common evaluating metric in the ML community.

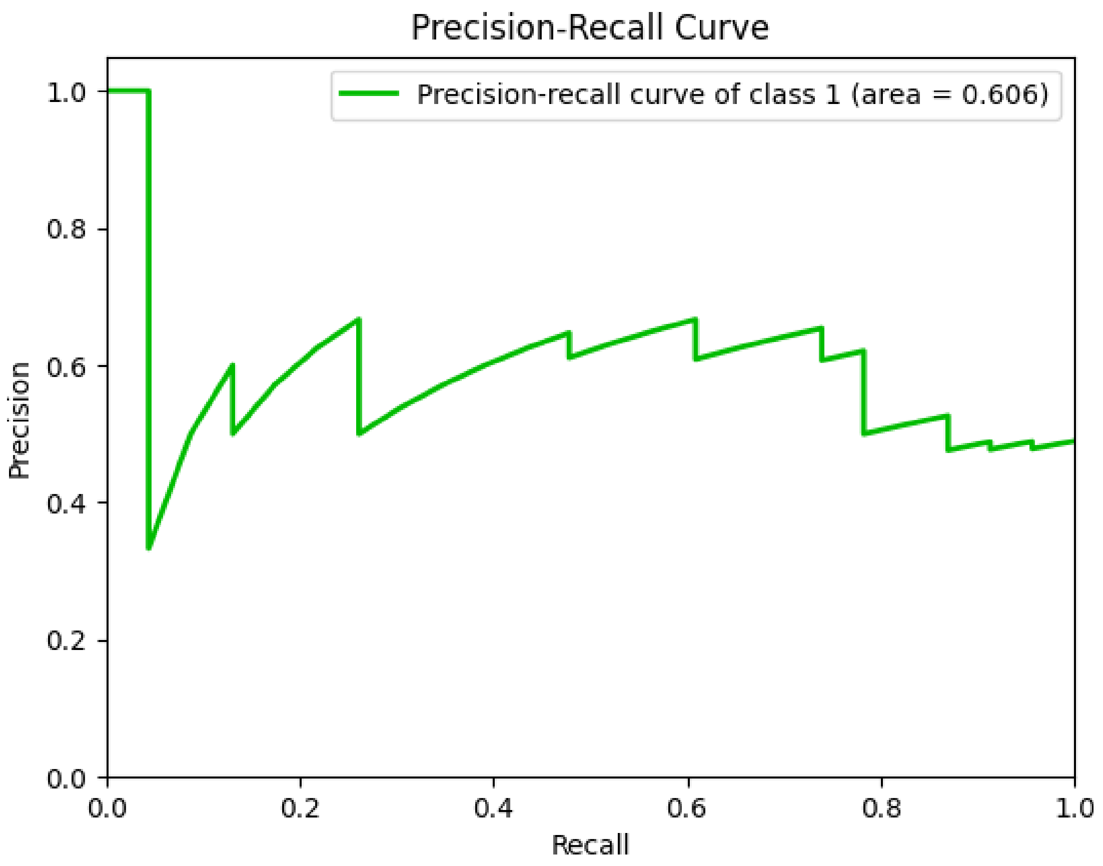

Precision-recall curve

Credit

AutoPeptideML has been developed and is maintained by Raul Fernandez-Diaz PhD Student at UCD and IBM Research under the supervision of Denis C. Shields (UCD Conway Institute and School of Medicine) and Thanh Lam Hoang (IBM Research).

If you have found the tool useful consider citing out paper:
