# Supplementary material for: AutoPeptideML: a study on how to build more trustworthy peptide bioactivity predictors
Source: Bioinformatics. 2024 Sep 18;40(9):btae555. doi: 10.1093/bioinformatics/btae555 (PMC11438549; doi:10.1093/bioinformatics/btae555)
Supplement: btae555_Supplementary_Data [file btae555_supplementary_data.zip › AutoPeptideML_OUP_SI_final.pdf]

A. APML-Peptipedia

The original Peptipedia database integrates information from 30 peptide bioactivity databases collecting almost 97,331 bioactive peptides labelled with 128 bioactivities (version `29_03_2023`). APML-Peptipedia is the result of removing all sequences with non-standard residues or without any known bioactivity and contains 92,092 peptides (see Supplementary). Figure 1 describes the distribution of the physicochemical properties of the peptides comprising APML-Peptipedia.

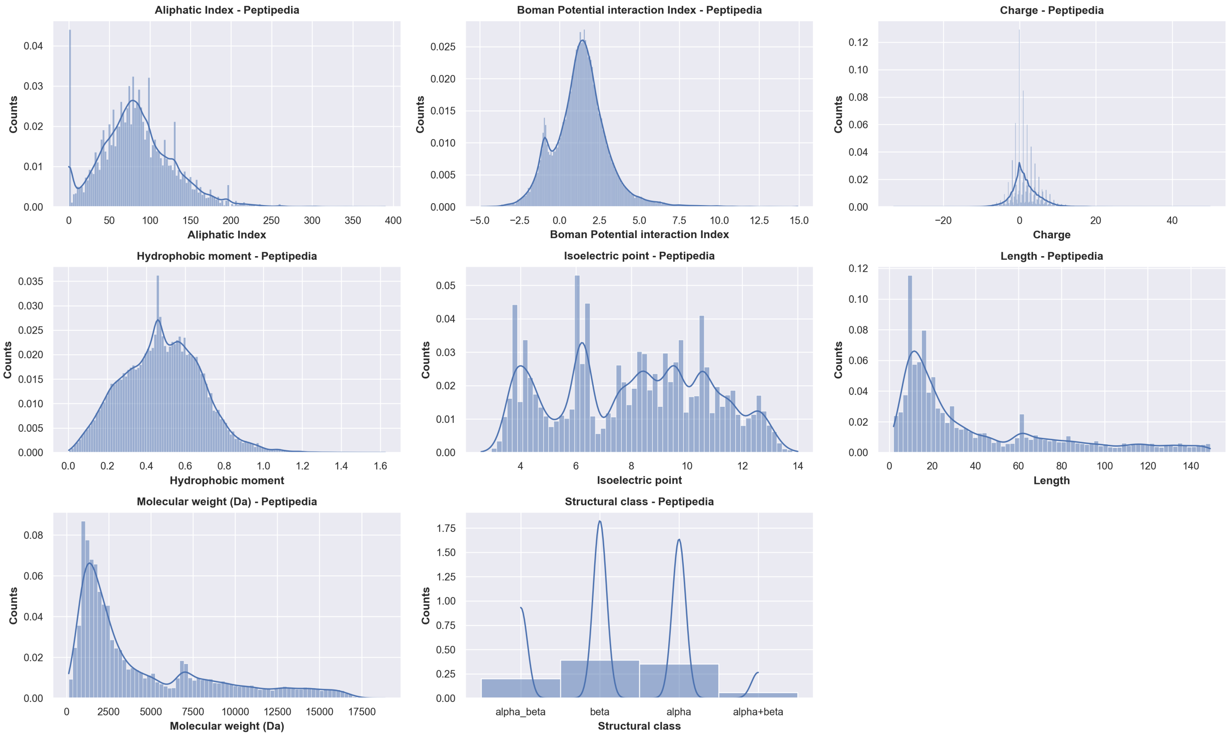

Fig. 1. Histograms describing the physicochemical properties of the Antiviral dataset. Curves represent the kernel density estimators of the different underlying distributions.

B. Search for Negative Peptides

Table 1 compiles the bioactivity tags excluded from the negative set when building the "NegSearch" datasets. The meaning behind these tags can be further expanded in the original publication (Quiroz et al., 2021).

Table 1. Overlapping classes excluded from the negative set for each of the benchmark datasets.

Dataset	Overlapping bioactivities
Antibacterial	`Antibacterial/antibiotic`
ACE inhibitor	`Blood_pressure,Blood_processes,Vasodilator,Vascular`
Anticancer	`Anicancer,Cytotoxic,Antitumour`
Antifungal	`Antifungal`
Antimalarial	`Antimalarial/antiplasmodial`
Antimicrobial	`Antimicrobial`
Antioxidant	`Antioxidant`
Antiparasitic	`Antiparasitic`
Antiviral	`Antiviral`
Blood-brain barrier	`Neuropeptide,Blood-brain_barrier_crossing`
DPPIV inhibitor	`Diabetic`
Anti-MRSA	`Antibacterial/antibiotic`
Neuropeptide	`Neuropeptide`
Quorum sensing	`Quorum_sensing`
Toxicity	`Cytotoxic,Neurotoxin,Toxic,Toxins`
Tumour T-cell antigens	`Immunological_activity`

C. Default hyperparameter search space

Table 2 describes the hyperparameter space defined for all experiments using the “Optimised ML ensemble”.

Table 2. Default hyperparameter search space for the ensemble used throughout the paper.

Model	Trials	Hyperparameter search space			
		**Name**	**Type**	**Range**	**Log-scale**
KNN	10	K	integer	1-30	No
		Weights	categorical	uniform or distance	No
RFC	10	Max depth	integer	2-20	No
		Number of estimators	integer	10-100	No
LightGBM	10	Max depth	integer	1-30	Yes
		Number of leaves	integer	5-50	Yes
		Learning rate	float	10e-3 - 0.3	Yes

D. UniDL4BioPep model architecture

UniDL4BioPep (Du et al., 2023) computes the peptide-level representations using ESM2-8M in the same way as described in Methods, by averaging across all residue-level representations. It then uses a 1D-convolutional neural network (1D-CNN) to make the predictions. The architecture for this 1D-CNN are fixed and are described in Table 3.

Table 3. UniDL4BioPep architecture.

Layer	Type of layer	Input	Size	Output
1	Conv1D	320	32	32 × 320
2	MaxPool	32 × 320	~	32 × 160
3	Flatten	32 × 160	~	5,120
4	Dense	5,120	64	64
5	Output	64	2	2

E. Dataset diversity

The effect of the homology-based diversity of the training and testing subsets is represented in Table S4. Figures S2-S17 describe the physicochemical composition of all the datasets, showing the differences between positive and negative peptides.

Table 4. Number of connected components clusters per dataset. Original: refers to the dataset with original set of negatives; New: refers to the datasets with the new negatives.

Dataset	Original Training	NegSearch+HP Training	Original Test	NegSearch+HP Test
Antibacterial	1,339	3,288	6,642	2,229
Inhibitor of ACE enzyme	1,570	421	1,749	763
Anticancer	372	344	440	179
Antifungal	297	348	940	341
Antimalarial	173	55	1,389	278
Antimicrobial	1,087	2,276	9,880	6,827
Antioxidant	694	174	1,119	240
Antiparasitic	234	120	1,362	77
Antiviral	1,690	1,174	3,242	1,003
Brain-blood barrier	173	47	157	36
Inhibitor of DPPIV enzyme	1,014	265	868	261
Anti-MRSA	100	59	752	195
Neuropeptide	2,331	968	2,601	816
Quorum sensing	301	87	236	38
Toxicity	754	717	811	334
Tumor T-cell antigen	885	236	850	195

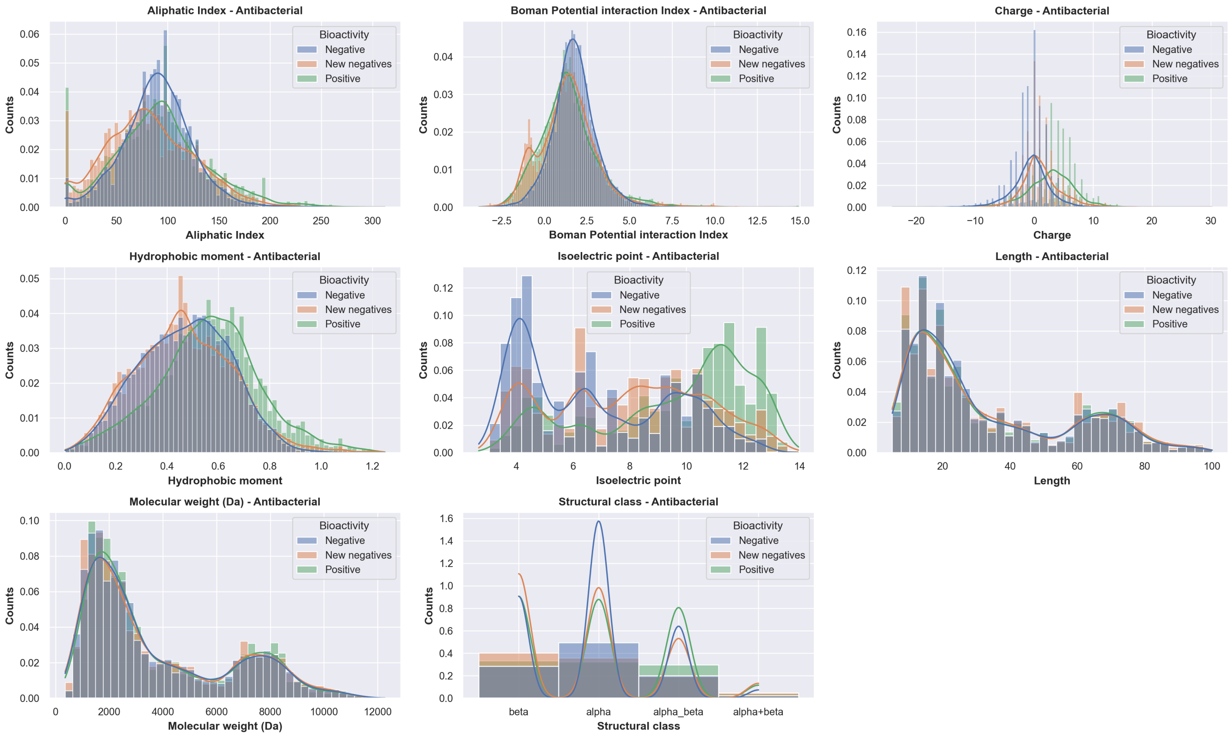

Fig. 2. Histograms describing the physicochemical properties of the Antibacterial dataset. Curves represent the kernel density estimators of the different underlying distributions.

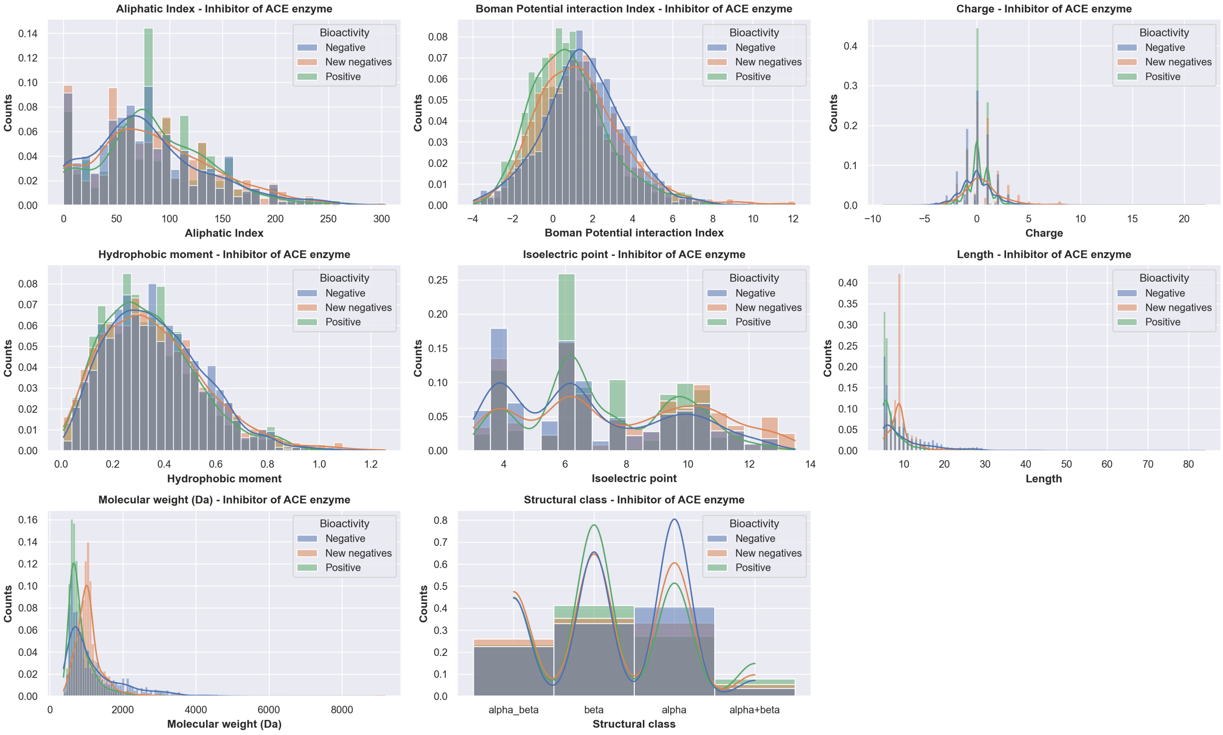

Fig. 3. Histograms describing the physicochemical properties of the ACE inhibitor dataset. Curves represent the kernel density estimators of the different underlying distributions.

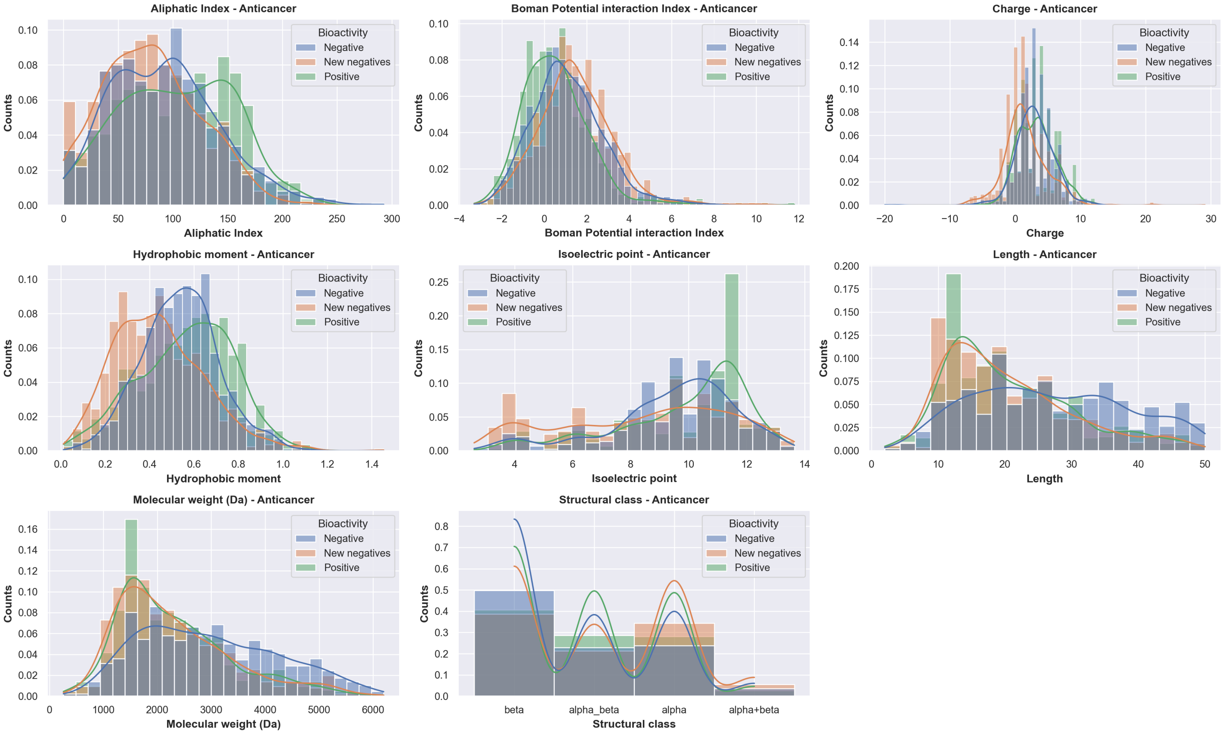

Fig. 4. Histograms describing the physicochemical properties of the Anticancer dataset. Curves represent the kernel density estimators of the different underlying distributions.

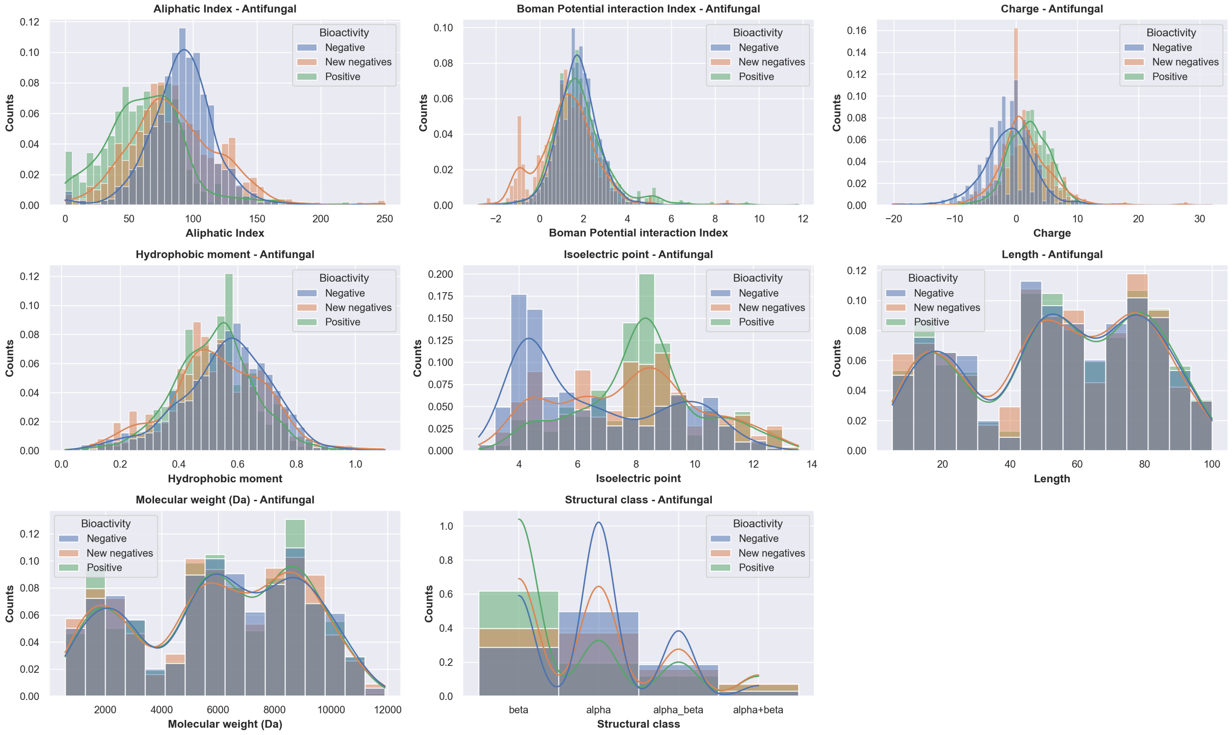

Fig. 5. Histograms describing the physicochemical properties of the Antifungal dataset. Curves represent the kernel density estimators of the different underlying distributions.

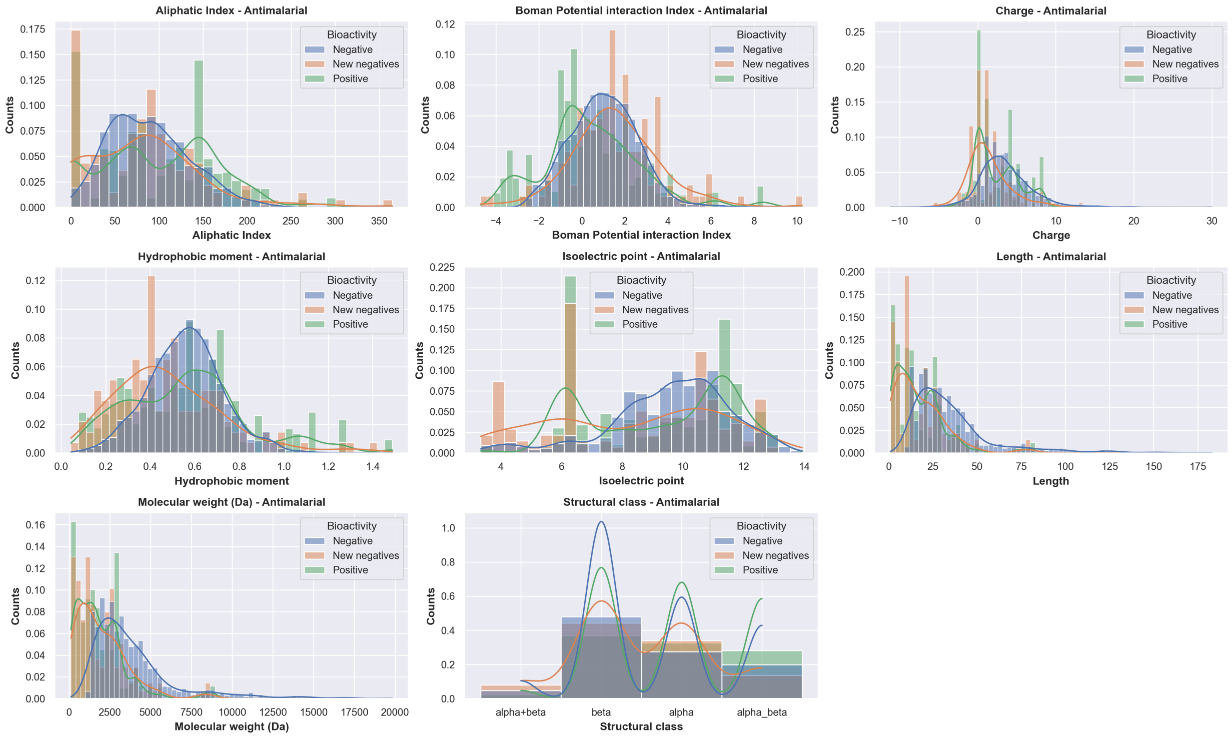

Fig. 6. Histograms describing the physicochemical properties of the Antimalarial dataset. Curves represent the kernel density estimators of the different underlying distributions.

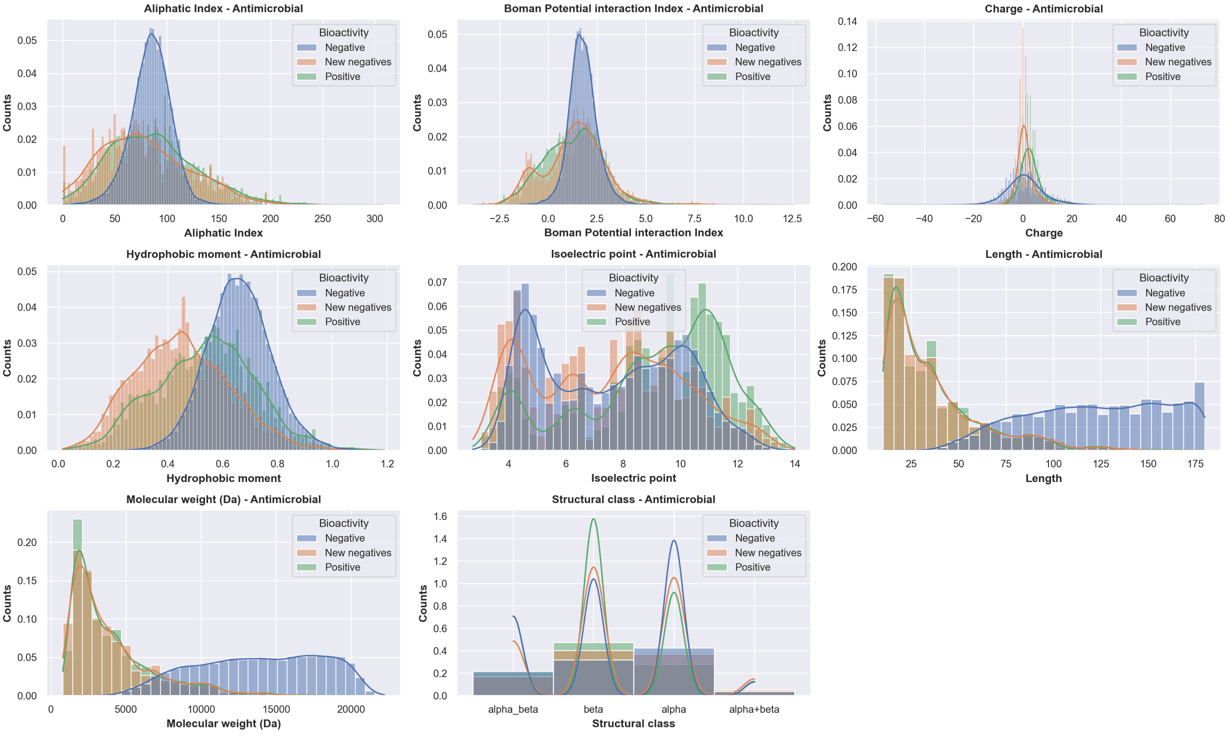

Fig. 7. Histograms describing the physicochemical properties of the Antimicrobial dataset. Curves represent the kernel density estimators of the different underlying distributions.

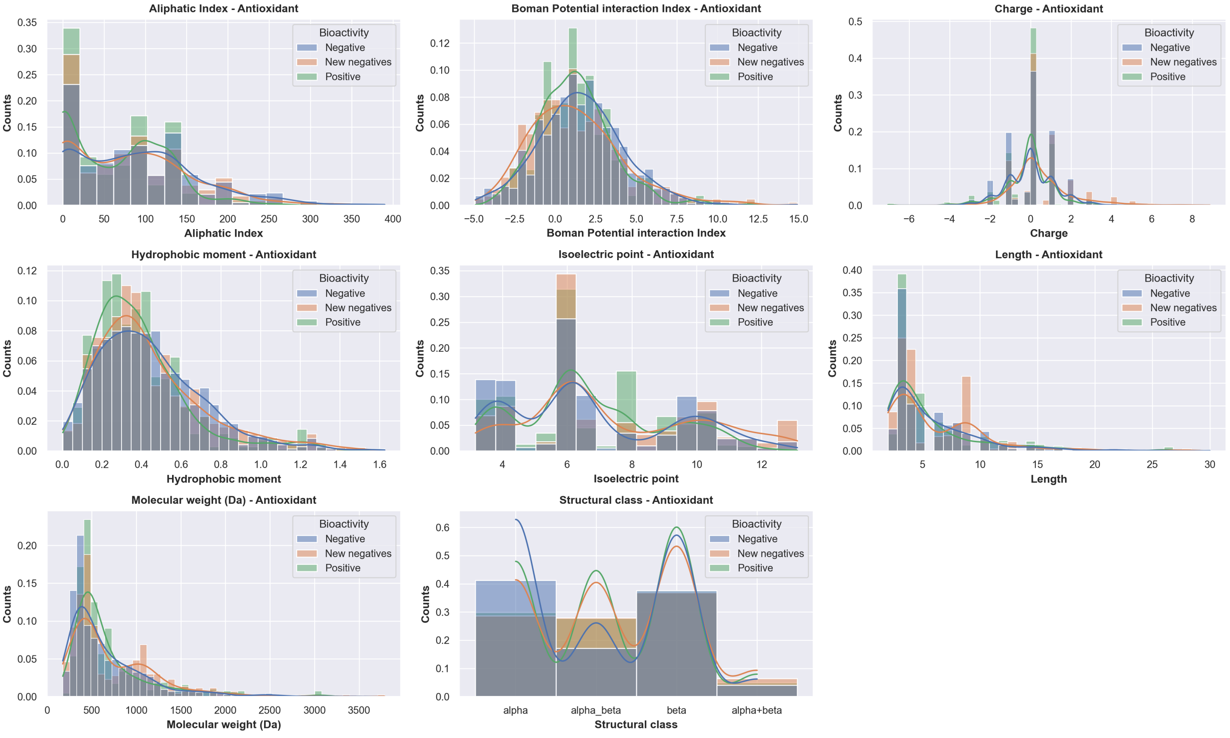

Fig. 8. Histograms describing the physicochemical properties of the Antioxidant dataset. Curves represent the kernel density estimators of the different underlying distributions.

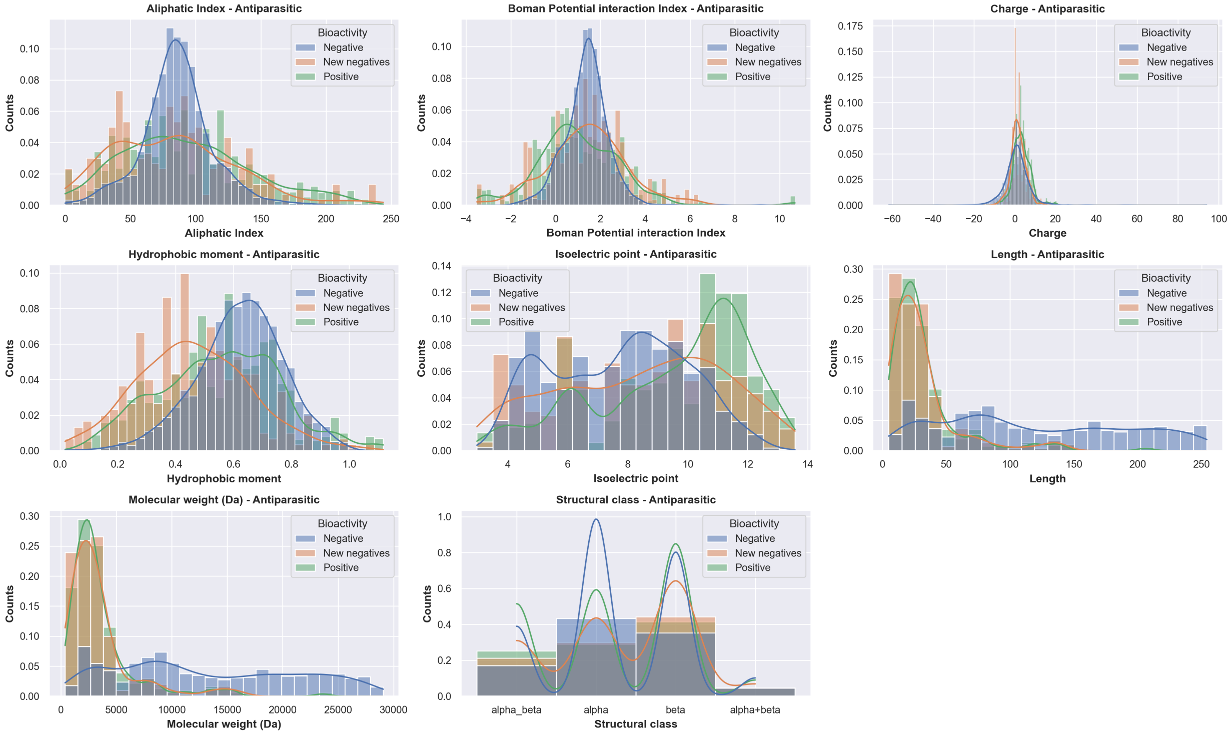

Fig. 9. Histograms describing the physicochemical properties of the Antiparasitic dataset. Curves represent the kernel density estimators of the different underlying distributions.

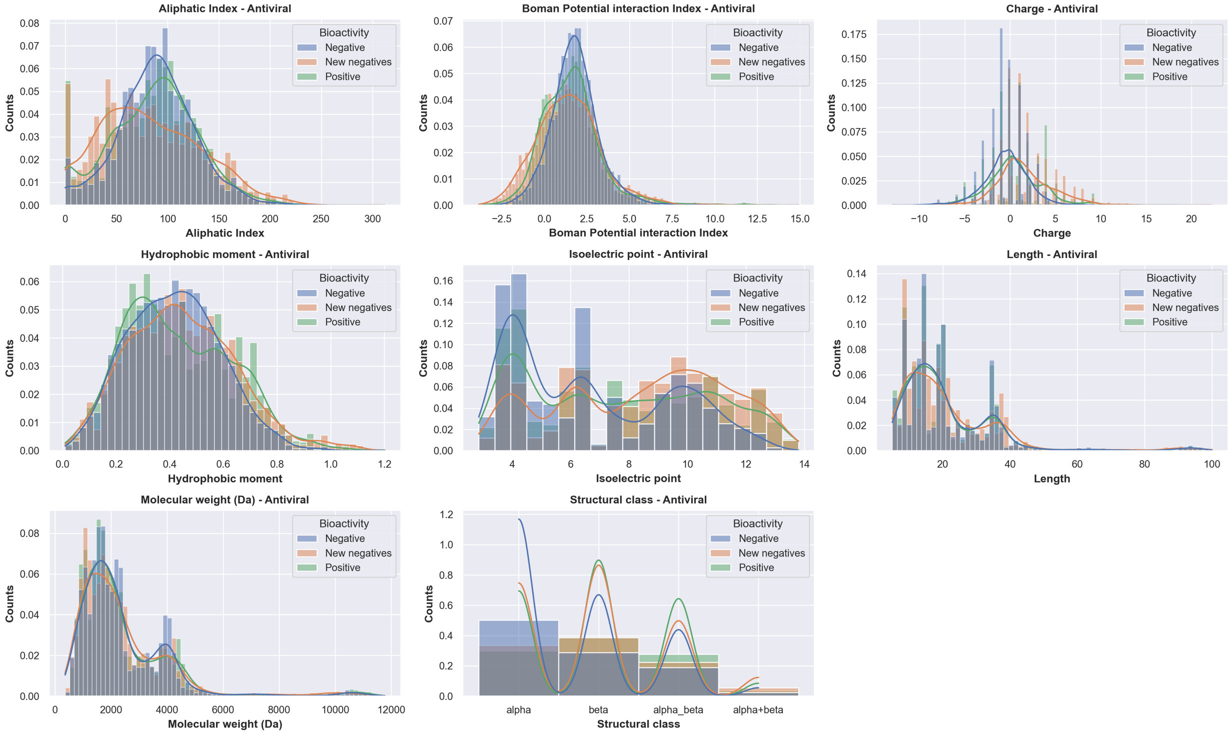

Fig. 10. Histograms describing the physicochemical properties of the Antiviral dataset. Curves represent the kernel density estimators of the different underlying distributions.

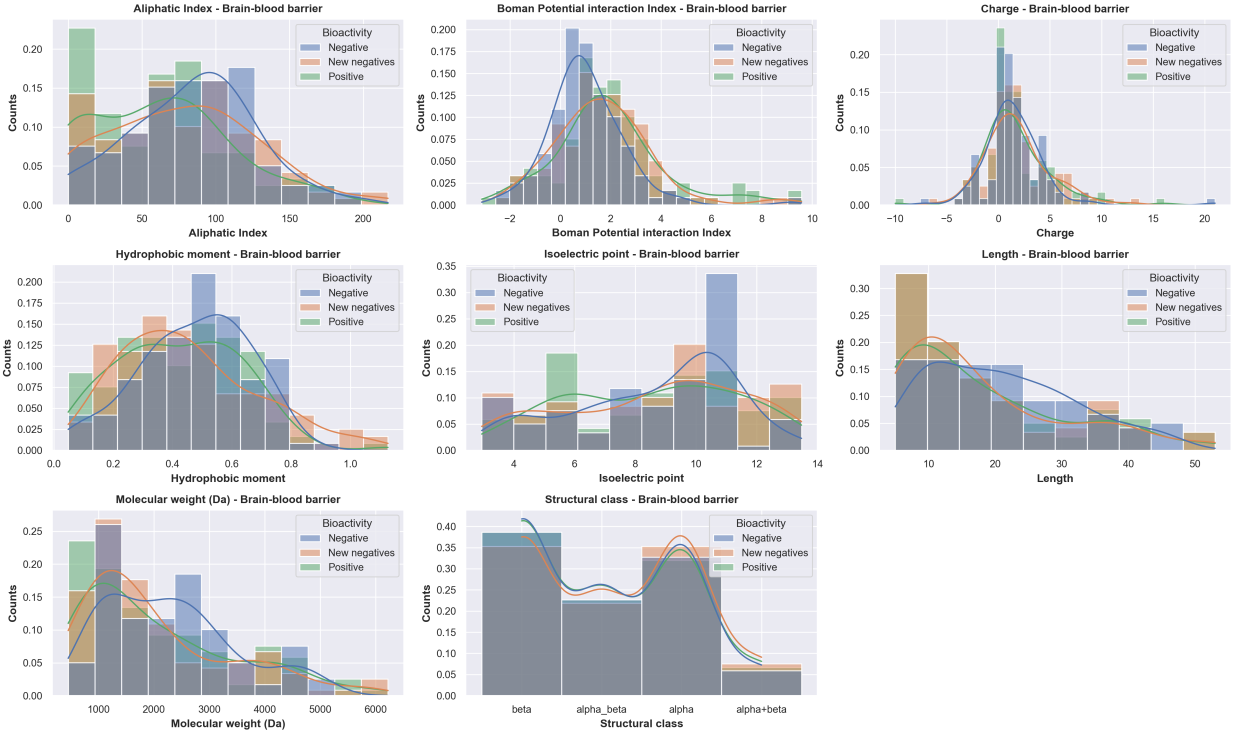

Fig. 11. Histograms describing the physicochemical properties of the Brain-blood barrier crossing dataset. Curves represent the kernel density estimators of the different underlying distributions.

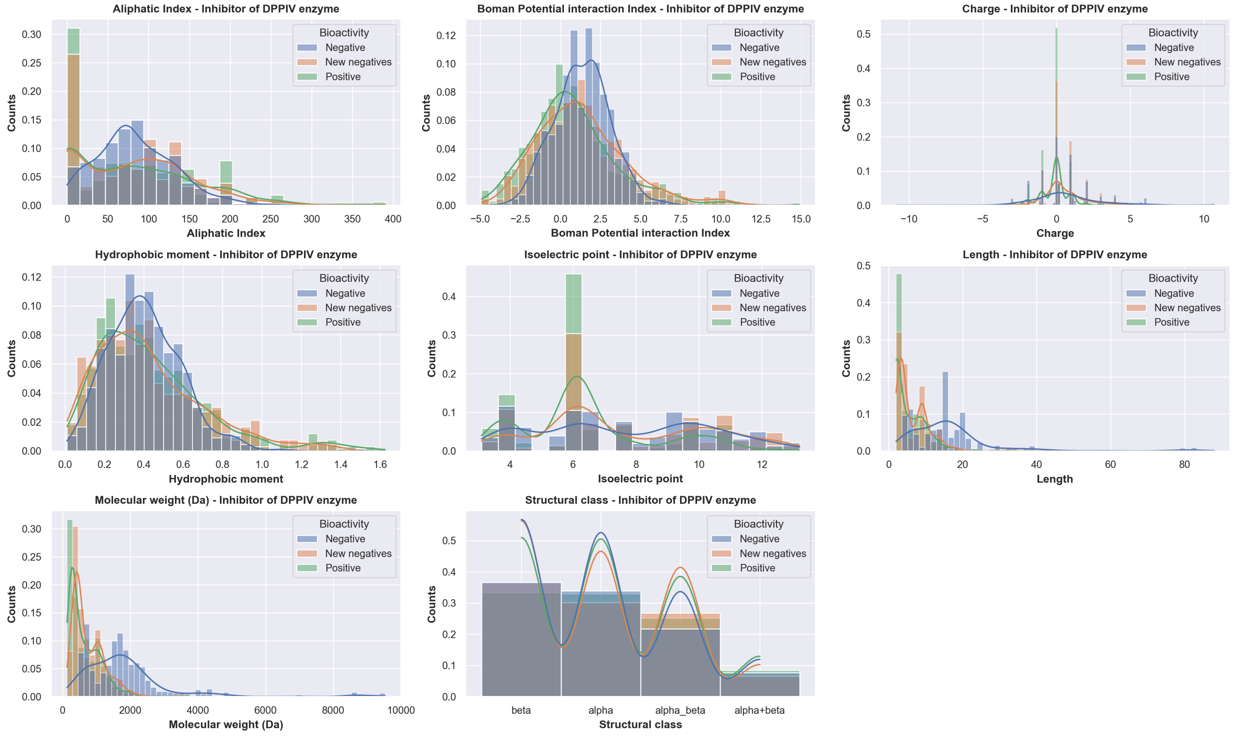

Fig. 12. Histograms describing the physicochemical properties of the DPPIV inhibitor dataset. Curves represent the kernel density estimators of the different underlying distributions.

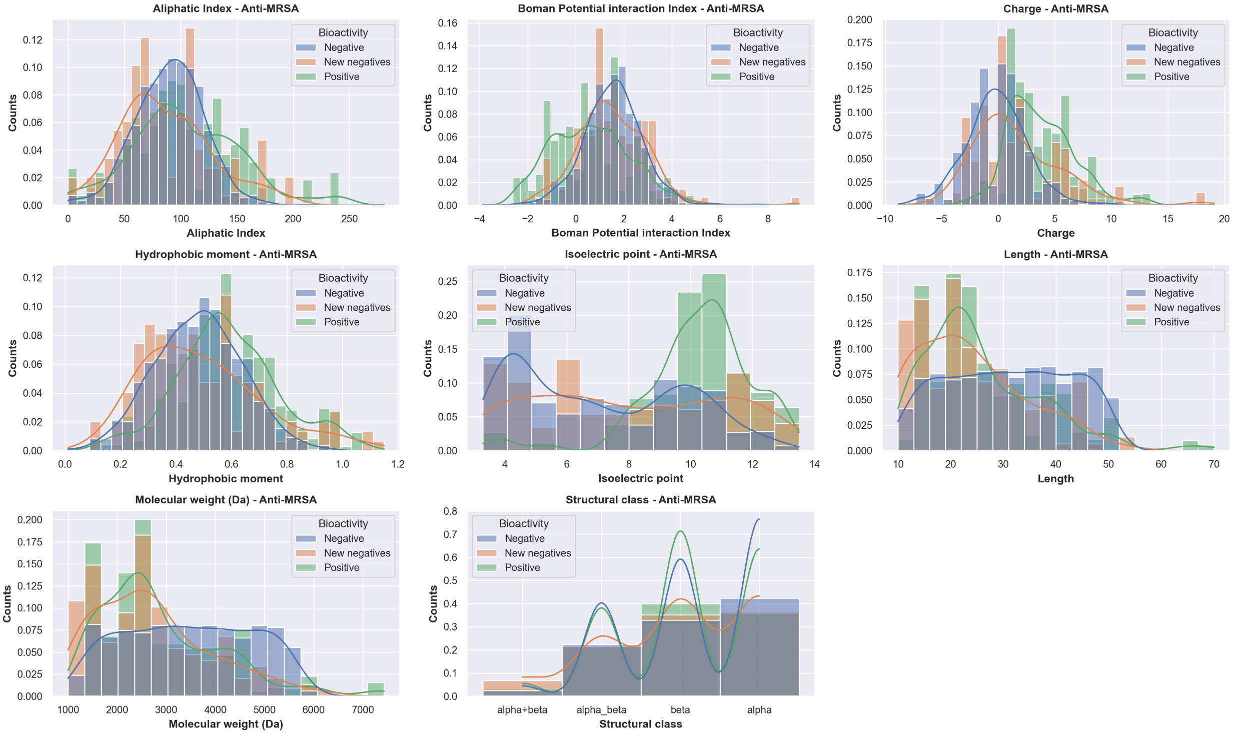

Fig. 13. Histograms describing the physicochemical properties of the Anti-MRSA dataset. Curves represent the kernel density estimators of the different underlying distributions.

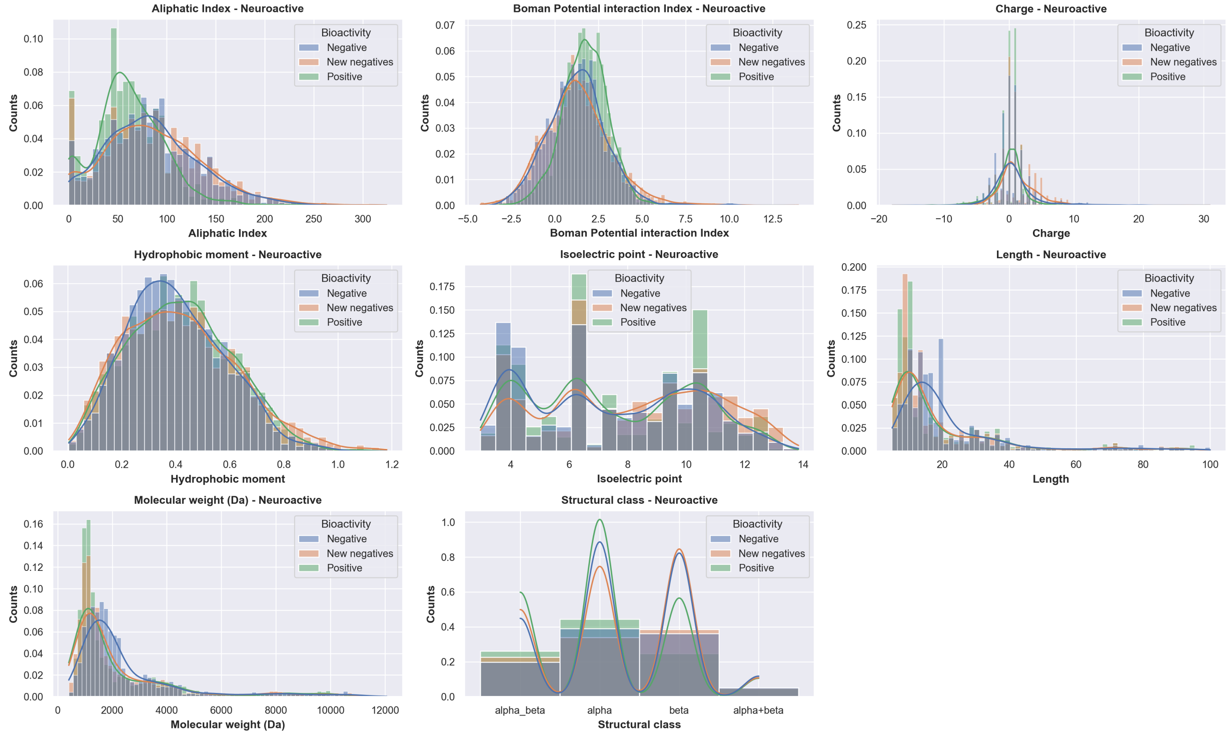

Fig. 14. Histograms describing the physicochemical properties of the Neuropeptide dataset. Curves represent the kernel density estimators of the different underlying distributions.

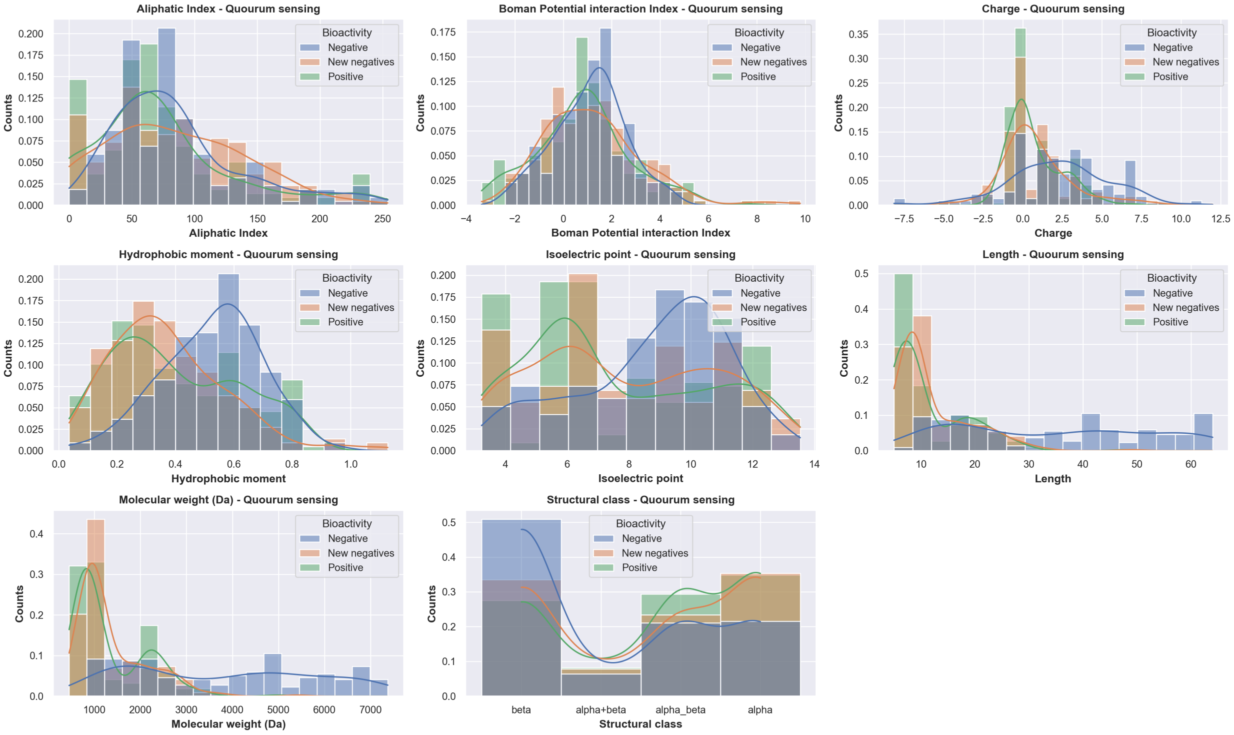

Fig. 15. Histograms describing the physicochemical properties of the Quorum sensing dataset. Curves represent the kernel density estimators of the different underlying distributions.

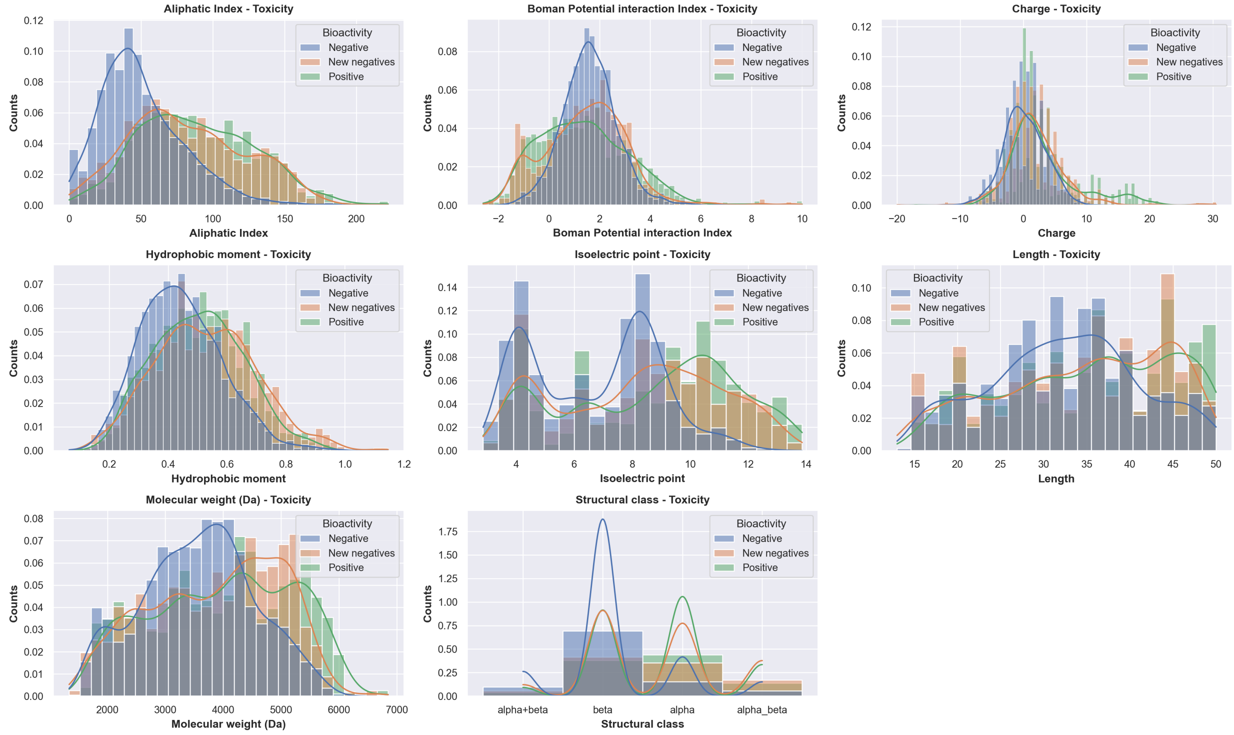

Fig. 16. Histograms describing the physicochemical properties of the Toxicity dataset. Curves represent the kernel density estimators of the different underlying distributions.

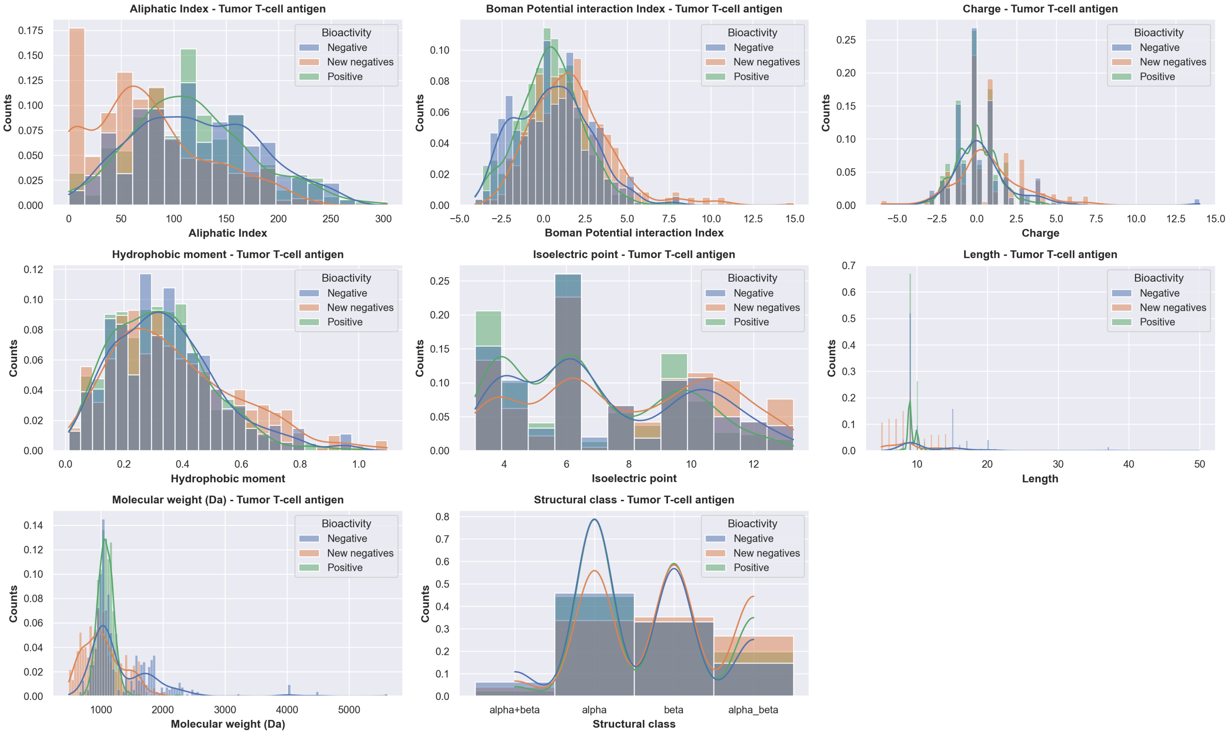

Fig. 17. Histograms describing the physicochemical properties of the Tumor T-cell antigen dataset. Curves represent the kernel density estimators of the different underlying distributions.

F. Metrics for model performance

This sections contains alternative metrics for all experimental results shown throughout the text.

Evaluation of AutoPeptideML's dataset construction modules

This subsection focuses on expanding on Figure 3. It includes Tables S5.

Table 5. Alternative metrics for the evaluation of AutoPeptideML's dataset construction modules. Errors represent the standard error of the mean across three different runs. Original: Original benchmark; NegSearch: Dataset with new negative peptides; HP: Homology-based dataset partitioning module; ACC: Accuracy; MCC: Mathew's correlation coeficient; AUROC: Area Under the ROC curve; F1: F1 score; BBBC: Brain-blood barrier crossing; TTCA: Tumor T-cell antigens.

Dataset	Metric	Original	NegSearch	NegSearch+HP
Antibacterial	ACC	0.9289 ± 0.0009	0.70 ± 0.02	0.717 ± 0.007
	MCC	0.858 ± 0.001	0.42 ± 0.02	0.45 ± 0.01
	AUROC	0.9770 ± 0.0006	0.767 ± 0.008	0.790 ± 0.005
	F1	0.926 ± 0.001	0.65 ± 0.03	0.67 ± 0.01
ACE inhibitor	ACC	0.855 ± 0.007	0.80 ± 0.01	0.783 ± 0.007
	MCC	0.71 ± 0.01	0.61 ± 0.03	0.57 ± 0.01
	AUROC	0.922 ± 0.008	0.87 ± 0.01	0.85 ± 0.01
	F1	0.856 ± 0.007	0.80 ± 0.01	0.786 ± 0.006
Anticancer	ACC	0.83 ± 0.05	0.63 ± 0.01	0.65 ± 0.01
	MCC	0.7 ± 0.1	0.30 ± 0.03	0.33 ± 0.01
	AUROC	0.88 ± 0.04	0.687 ± 0.006	0.707 ± 0.009
	F1	0.83 ± 0.05	0.55 ± 0.01	0.57 ± 0.03
Antifungal	ACC	0.947 ± 0.002	0.61 ± 0.01	0.594 ± 0.009
	MCC	0.895 ± 0.005	0.0.26 ± 0.02	0.20 ± 0.02
	AUROC	0.991 ± 0.002	0.63 ± 0.02	0.62 ± 0.02
	F1	0.944 ± 0.002	0.51 ± 0.02	0.49 ± 0.02
Antimalarial	ACC	0.984 ± 0.002	0.66 ± 0.02	0.69 ± 0.03
	MCC	0.89 ± 0.03	0.33 ± 0.04	0.39 ± 0.06
	AUROC	0.98 ± 0.01	0.74 ± 0.03	0.752 ± 0.003
	F1	0.90 ± 0.03	0.66 ± 0.04	0.0692 ± 0.009
Antimicrobial	ACC	0.953 ± 0.001	0.656 ± 0.008	0.645 ± 0.004
	MCC	0/887 ± 0.003	0.32 ± 0.01	0.292 ± 0.009
	AUROC	0.9860 ± 0.0008	0.736 ± 0.008	0.723 ± 0.007
	F1	0.919 ± 0.002	0.60 ± 0.02	0.628 ± 0.005
Antioxidant	ACC	0.83 ± 0.02	0.66 ± 0.02	0.63 ± 0.03
	MCC	0.67 ± 0.02	0.34 ± 0.05	0.26 ± 0.06
	AUROC	0.897 ± 0.002	0.71 ± 0.03	0.71 ± 0.03
	F1	0.814 ± 0.008	0.67 ± 0.04	0.64 ± 0.03
Antiparasitic	ACC	0.76 ± 0.02	0.700 ± 0.03	0.71 ± 0.04
	MCC	0.56 ± 0.02	0.40 ± 0.06	0.41 ± 0.07
	AUROC	0.930 ± 0.004	0.75 ± 0.03	0.76 ± 0.04
	F1	0.70 ± 0.03	0.69 ± 0.03	0.71 ± 0.03
Antiviral	ACC	0.828 ± 0.005	0.74 ± 0.02	0.760 ± 0.005
	MCC	0.659 ± 0.009	0.49 ± 0.05	0.520 ± 0.009
	AUROC	0.898 ± 0.005	0.82 ± 0.01	0.840 ± 0.005
	F1	0.821 ± 0.007	0.73 ± 0.03	0.764 ± 0.003
BBBC	ACC	0.80 ± 0.02	0.74 ± 0.02	0.54 ± 0.04
	MCC	0.60 ± 0.05	0.19 ± 0.06	0.08 ± 0.08
	AUROC	0.907 ± 0.008	0.65 ± 0.08	0.55 ± 0.06
	F1	0.79 ± 0.03	0.60 ± 0.03	0.0.56 ± 0.02
DPPIV inhibitor	ACC	0.83 ± 0.02	0.60 ± 0.03	0.74 ± 0.01
	MCC	0.67 ± 0.04	0.56 ± 0.04	0.47 ± 0.03
	AUROC	0.927 ± 0.002	0.84 ± 0.02	0.816 ± 0.008
	F1	0.83 ± 0.02	0.78 ± 0.02	0.73 ± 0.02
Anti-MRSA	ACC	0.998 ± 0.001	0.78 ± 0.02	0.60 ± 0.04
	MCC	0.993 ± 0.007	0.49 ± 0.03	0.41 ± 0.09
	AUROC	1.0000 ± 0.0000	0.82 ± 0.01	0.789 ± 0.009
	F1	0.994 ± 0.006	0.69 ± 0.02	0.65 ± 0.03
Neuropeptide	ACC	0.850 ± 0.002	0.853 ± 0.004	0.817 ± 0.007
	MCC	0.705 ± 0.003	0.708 ± 0.008	0.64 ± 0.01
	AUROC	0.937 ± 0.001	0.919 ± 0.002	0.908 ± 0.004
	F1	0.858 ± 0.002	0.852 ± 0.004	0.830 ± 0.05
Quorum sensing	ACC	0.908 ± 0.008	0.839 ± 0.007	0.82 ± 0.04
	MCC	0.82 ± 0.02	0.68 ± 0.01	0.65 ± 0.08
	AUROC	0.967 ± 0.005	0.934 ± 0.008	0.93 ± 0.02
	F1	0.908 ± 0.008	0.842 ± 0.005	0.83 ± 0.03
Toxicity	ACC	0.914 ± 0.004	0.684 ± 0.006	0.628 ± 0.003
	MCC	0.828 ± 0.009	0.39 ± 0.01	0.26 ± 0.006
	AUROC	0.9677 ± 0.0007	0.756 ± 0.001	0.736 ± 0.005
	F1	0.919 ± 0.004	0.727 ± 0.006	0.670 ± 0.004
TTCA	ACC	0.689 ± 0.009	0.900 ± 0.008	0.869 ± 0.009
	MCC	0.33 ± 0.02	0.80 ± 0.02	0.74 ± 0.02
	AUROC	0.70 ± 0.01	0.95 ± 0.02	0.93 ± 0.01
	F1	0.753 ± 0.008	0.899 ± 0.007	0.877 ± 0.007

Evaluation of different protein language models

This subsection focuses on expanding on Figure 3. It includes Tables S6-S7.

Table 6. Alternative metrics for the evaluation for different protein language models. Errors represent the standard error of the mean across three different runs. ACC: Accuracy; MCC: Mathew's correlation coeficient; AUROC: Area Under the ROC curve; F1: F1 score; BBBC: Brain-blood barrier crossing; TTCA: Tumor T-cell antigens.

Dataset	Metric	ESM2 8M	ESM2 35M	ESM2 150M	ESM2 650M	ESM1b 650M	ProtBERT	Prot-T5-XL	Prost-T5
Antibacterial	ACC	0.717 ± 0.007	0.720 ± 0.002	0.726 ± 0.002	0.719 ± 0.002	0.699 ± 0.005	0.711 ± 0.001	0.70 ± 0.01	0.67 ± 0.02
	MCC	0.45 ± 0.01	0.457 ± 0.005	0.465 ± 0.003	0.459 ± 0.005	0.42 ± 0.01	0.43 ± 0.02	0.41 ± 0.02	0.35 ± 0.04
	AUROC	0.790 ± 0.005	0.794 ± 0.003	0.799 ± 0.005	0.796 ± 0.003	0.793 ± 0.005	0.780 ± 0.007	0.787 ± 0.006	0.754 ± 0.009
	F1	0.67 ± 0.01	0.677 ± 0.002	0.689 ± 0.002	0.670 ± 0.005	0.65 ± 0.01	0.679 ± 0.01	0.639 ± 0.02	0.60 ± 0.04
ACE inhibitor	ACC	0.783 ± 0.007	0.79 ± 0.01	0.77 ± 0.01	0.776 ± 0.002	0.79 ± 0.02	0.72 ± 0.01	0.7561 ± 0.0008	0.7593 ± 0.007
	MCC	0.57 ± 0.1	0.58 ± 0.02	0.53 ± 0.03	0.552 ± 0.003	0.589 ± 0.03	0.44 ± 0.02	0.513 ± 0.001	0.52 ± 0.01
	AUROC	0.85 ± 0.01	0.85 ± 0.01	0.84 ± 0.01	0.854 ± 0.007	0.86 ± 0.02	0.78 ± 0.02	0.833 ± 0.005	0.83 ± 0.01
	F1	0.786 ± 0.006	0.79 ± 0.01	0.78 ± 0.01	0.776 ± 0.005	0.79 ± 0.01	0.72 ± 0.01	0.760 ± 0.004	0.764 ± 0.006
Anticancer	ACC	0.65 ± 0.01	0.65 ± 0.01	0.64 ± 0.01	0.0612 ± 0.009	0.616 ± 0.004	0.63 ± 0.01	0.62 ± 0.01	0.620 ± 0.007
	MCC	0.33 ± 0.02	0.33 ± 0.02	0.30 ± 0.03	0.26 ± 0.02	0.27 ± 0.01	0.28 ± 0.02	0.26 ± 0.03	0.25 ± 0.02
	AUROC	0.707 ± 0.009	0.73 ± 0.02	0.73 ± 0.01	0.73 ± 0.02	0.72 ± 0.02	0.71 ± 0.02	0.71 ± 0.01	0.680 ± 0.009
	F1	0.57 ± 0.03	0.57 ± 0.04	0.54 ± 0.05	0.47 ± 0.02	0.472 ± 0.007	0.56 ± 0.01	0.51 ± 0.03	0.546 ± 0.004
Antifungal	ACC	0.594 ± 0.009	0.62 ± 0.01	0.64 ± 0.01	0.651 ± 0.009	0.60 ± 0.02	0.62 ± 0.02	0.749 ± 0.007	0.611 ± 0.01
	MCC	0.20 ± 0.02	0.25 ± 0.04	0.28 ± 0.03	0.33 ± 0.02	0.22 ± 0.05	0.23 ± 0.04	0.31 ± 0.01	0.23 ± 0.03
	AUROC	0.62 ± 0.02	0.67 ± 0.01	0.71 ± 0.02	0.72 ± 0.01	0.69 ± 0.02	0.68 ± 0.03	0.72 ± 0.01	0.66 ± 0.2
	F1	0.49 ± 0.02	0.564 ± 0.009	0.587 ± 0.004	0.56 ± 0.02	0.517 ± 0.008	0.60 ± 0.02	0.59 ± 0.02	0.547 ± 0.009
Antimalarial	ACC	0.63 ± 0.03	0.63 ± 0.03	0.66 ± 0.04	0.72 ± 0.03	0.69 ± 0.02	0.648 ± 0.03	0.67 ± 0.02	0.70 ± 0.05
	MCC	0.39 ± 0.06	0.27 ± 0.06	0.32 ± 0.07	0.43 ± 0.07	0.38 ± 0.04	0.30 ± 0.05	0.33 ± 0.03	0.4 ± 0.1
	AUROC	0.752 ± 0.003	0.72 ± 0.02	0.72 ± 0.03	0.80 ± 0.01	0.74 ± 0.02	0.72 ± 0.04	0.70 ± 0.02	0.74 ± 0.03
	F1	0.692 ± 0.009	0.64 ± 0.04	0.66 ± 0.04	0.72 ± 0.03	0.68 ± 0.02	0.61 ± 0.04	0.62 ± 0.02	0.68 ± 0.06
Antimicrobial	ACC	0.645 ± 0.005	0.6494 ± 0.0009	0.649 ± 0.005	0.64 ± 0.1	0.638 ± 0.009	0.619 ± 0.004	0.659 ± 0.009	0.640 ± 0.006
	MCC	0.292 ± 0.009	0.300 ± 0.002	0.30 ± 0.01	0.29 ± 0.02	0.28 ± 0.02	0.239 ± 0.008	0.32 ± 0.02	0.28 ± 0.01
	AUROC	0.723 ± 0.007	0.722 ± 0.004	0.723 ± 0.004	0.731 ± 0.007	0.723 ± 0.007	0.682 ± 0.007	0.74 ± 0.01	0.737 ± 0.008
	F1	0.628 ± 0.005	0.632 ± 0.003	0.627 ± 0.005	0.62 ± 0.01	0.61 ± 0.01	0.617 ± 0.005	0.644 ± 0.009	0.621 ± 0.005
Antioxidant	ACC	0.63 ± 0.03	0.632 ± 0.008	0.62 ± 0.03	0.63 ± 0.03	0.66 ± 0.03	0.59 ± 0.04	0.068 ± 0.03	0.65 ± 0.03
	MCC	0.27 ± 0.06	0.29 ± 0.02	0.25 ± 0.02	0.27 ± 0.06	0.31 ± 0.06	0.18 ± 0.07	0.37 ± 0.06	0.31 ± 0.05
	AUROC	0.71 ± 0.03	0.71 ± 0.02	0.69 ± 0.04	0.70 ± 0.02	0.72 ± 0.03	0.66 ± 0.04	0.74 ± 0.05	0.70 ± 0.03
	F1	0.64 ± 0.03	0.66 ± 0.01	0.63 ± 0.03	0.66 ± 0.03	0.65 ± 0.04	0.59 ± 0.04	0.69 ± 0.04	0.66 ± 0.03
Antiparasitic	ACC	0.71 ± 0.03	0.70 ± 0.02	0.69 ± 0.02	0.700 ± 0.01	0.70 ± 0.03	0.71 ± 0.04	0.714 ± 0.003	0.69 ± 0.02
	MCC	0.41 ± 0.07	0.41 ± 0.04	0.37 ± 0.04	0.40 ± 0.02	0.41 ± 0.06	0.43 ± 0.07	0.428 ± 0.006	0.37 ± 0.03
	AUROC	0.76 ± 0.04	0.77 ± 0.03	0.77 ± 0.03	0.79 ± 0.02	0.78 ± 0.04	0.80 ± 0.04	0.79 ± 0.01	0.77 ± 0.02
	F1	0.71 ± 0.03	0.70 ± 0.02	0.716 ± 0.004	0.715 ± 0.004	0.71 ± 0.02	0.73 ± 0.03	0.715 ± 0.005	0.69 ± 0.02
Antiviral	ACC	0.760 ± 0.005	0.739 ± 0.003	0.737 ± 0.004	0.749 ± 0.008	0.745 ± 0.008	0.69 ± 0.02	0.747 ± 0.009	0.710 ± 0.007
	MCC	0.520 ± 0.009	0.470 ± 0.005	0.474 ± 0.008	0.50 ± 0.02	0.49 ± 0.02	0.38 ± 0.03	0.50 ± 0.02	0.42 ± 0.01
	AUROC	0.840 ± 0.005	0.811 ± 0.003	0.819 ± 0.002	0.825 ± 0.005	0.821 ± 0.006	0.77 ± 0.01	0.831 ± 0.05	0.80 ± 0.01
	F1	0.764 ± 0.003	0.745 ± 0.002	0.742 ± 0.004	0.760 ± 0.006	0.751 ± 0.008	0.70 ± 0.01	0.749 ± 0.007	0.712 ± 0.007
BBBC	ACC	0.54 ± 0.04	0.61 ± 0.05	0.56 ± 0.01	0.60 ± 0.01	0.62 ± 0.02	0.60 ± 0.03	0.72 ± 0.03	0.60 ± 0.09
	MCC	0.08 ± 0.08	0.22 ± 0.1	0.12 ± 0.03	0.21 ± 0.02	0.25 ± 0.04	0.21 ± 0.05	0.43 ± 0.05	0.19 ± 0.2
	AUROC	0.55 ± 0.06	0.58 ± 0.07	0.627 ± 0.006	0.64 ± 0.03	0.66 ± 0.03	0.64 ± 0.04	0.74 ± 0.02	0.64 ± 0.08
	F1	0.56 ± 0.02	0.61 ± 0.04	0.55 ± 0.5	0.60 ± 0.03	0.64 ± 0.02	0.57 ± 0.03	0.72 ± 0.03	0.59 ± 0.09
DPPIV inhibitor	ACC	0.75 ± 0.01	0.76 ± 0.02	0.731 ± 0.003	0.766 ± 0.008	0.76 ± 0.01	0.75 ± 0.01	0.77 ± 0.01	0.757 ± 0.007
	MCC	0.47 ± 0.03	0.52 ± 0.04	0.462 ± 0.007	0.53 ± 0.02	0.52 ± 0.02	0.50 ± 0.02	0.54 ± 0.03	0.52 ± 0.01
	AUROC	0.815 ± 0.008	0.84 ± 0.01	0.81 ± 0.1	0.834 ± 0.006	0.83 ± 0.01	0.82 ± 0.02	0.860 ± 0.006	0.836 ± 0.008
	F1	0.73 ± 0.02	0.76 ± 0.02	0.733 ± 0.007	0.77 ± 0.01	0.75 ± 0.02	0.75 ± 0.02	0.77 ± 0.02	0.75 ± 0.01
Anti-MRSA	ACC	0.69 ± 0.04	0.73 ± 0.01	0.64 ± 0.03	0.69 ± 0.04	0.68 ± 0.02	0.71 ± 0.04	0.66 ± 0.03	0.71 ± 0.03
	MCC	0.41 ± 0.09	0.48 ± 0.01	0.31 ± 0.08	0.42 ± 0.09	0.41 ± 0.06	0.43 ± 0.09	0.34 ± 0.07	0.44 ± 0.05
	AUROC	0.779 ± 0.009	0.81 ± 0.2	0.727 ± 0.005	0.81 ± 0.02	0.79 ± 0.03	0.80 ± 0.04	0.74 ± 0.03	0.76 ± 0.04
	F1	0.65 ± 0.03	0.70 ± 0.02	0.54 ± 0.04	0.60 ± 0.07	0.622 ± 0.002	0.685 ± 0.05	0.61 ± 0.05	0.67 ± 0.04
Neuropeptide	ACC	0.817 ± 0.007	0.82 ± 0.02	0.81 ± 0.01	0.833 ± 0.01	0.829 ± 0.007	0.77 ± 0.01	0.823 ± 0.003	0.78 ± 0.02
	MCC	0.64 ± 0.01	0.65 ± 0.03	0.63 ± 0.02	0.67 ± 0.02	0.66 ± 0.01	0.55 ± 0.02	0.654 ± 0.005	0.58 ± 0.03
	AUROC	0.908 ± 0.004	0.90 ± 0.01	0.90 ± 0.01	0.918 ± 0.008	0.905 ± 0.007	0.86 ± 0.01	0.912 ± 0.006	0.865 ± 0.01
	F1	0.830 ± 0.005	0.83 ± 0.01	0.82 ± 0.01	0.84 ± 0.01	0.839 ± 0.005	0.786 ± 0.009	0.836 ± 0.002	0.80 ± 0.01
Quorum sensing	ACC	0.82 ± 0.04	0.82 ± 0.03	0.82 ± 0.01	0.84 ± 0.01	0.85 ± 0.01	0.75 ± 0.04	0.831 ± 0.004	0.83 ± 0.03
	MCC	0.65 ± 0.08	0.64 ± 0.05	0.65 ± 0.02	0.67 ± 0.02	0.71 ± 0.03	0.51 ± 0.08	0.686 ± 0.003	0.66 ± 0.05
	AUROC	0.93 ± 0.02	0.92 ± 0.02	0.920 ± 0.006	0.938 ± 0.008	0.93 ± 0.02	0.85 ± 0.04	0.934 ± 0.004	0.90 ± 0.02
	F1	0.83 ± 0.03	0.82 ± 0.02	0.823 ± 0.003	0.836 ± 0.007	0.85 ± 0.02	0.76 ± 0.03	0.842 ± 0.001	0.84 ± 0.02
Toxicity	ACC	0.63 ± 0.01	0.627 ± 0.007	0.629 ± 0.003	0.63 ± 0.02	0.638 ± 0.007	0.62 ± 0.01	0.68 ± 0.01	0.640 ± 0.002
	MCC	0.264 ± 0.006	0.26 ± 0.01	0.264 ± 0.007	0.28 ± 0.03	0.28 ± 0.02	0.25 ± 0.03	0.36 ± 0.02	0.288 ± 0.004
	AUROC	0.736 ± 0.005	0.726 ± 0.009	0.721 ± 0.003	0.74 ± 0.01	0.74 ± 0.01	0.715 ± 0.009	0.793 ± 0.005	0.733 ± 0.004
	F1	0.670 ± 0.004	0.672 ± 0.005	0.666 ± 0.002	0.68 ± 0.02	0.674 ± 0.006	0.651 ± 0.01	0.706 ± 0.009	0.679 ± 0.004
TTCA	ACC	0.869 ± 0.009	0.86 ± 0.01	0.85 ± 0.01	0.87 ± 0.01	0.86 ± 0.01	0.82 ± 0.02	0.85 ± 0.02	0.8386 ± 0.02
	MCC	0.74 ± 0.02	0.72 ± 0.02	0.71 ± 0.02	0.74 ± 0.03	0.73 ± 0.02	0.64 ± 0.04	0.70 ± 0.03	0.74 ± 0.05
	AUROC	0.93 ± 0.01	0.92 ± 0.02	0.92 ± 0.01	0.926 ± 0.008	0.929 ± 0.008	0.88 ± 0.02	0.92 ± 0.02	0.93 ± 0.0.009
	F1	0.877 ± 0.007	0.87 ± 0.01	0.86 ± 0.01	0.87 ± 0.01	0.87 ± 0.01	0.83 ± 0.01	0.86 ± 0.01	0.8884 ± 0.02

Comparison of training strategies on original datasets

This subsection contains alternative metrics for the results in Figure 4.A. Figures S18-S35 represent the ROC curves corresponding to one of the three experiments. Table S8 contains alternative metrics for the comparison of training strategies on the original datasets.

Alternative metrics for the comparison of training strategies in the new datasets.

This subsection reflects the results in Figure 4.B. It includes Table S9.

Table 7. Metrics for one-hot encoding.

Dataset	MCC
Antibacterial	0.293 ± 0.007
ACE inhibitor	0.40 ± 0.06
Anticancer	0.270 ± 0.02
Antifungal	0.06 ± 0.03
Antimalarial	0.32 ± 0.08
Antimicrobial	0.076 ± 0.007
Antioxidant	0.239 ± 0.02
Antiparasitic	0.16 ± 0.04
Antiviral	0.385 ± 0.04
BBBC	0.39 ± 0.01
DPPIV inhibitor	0.40 ± 0.05
Anti-MRSA	0.32 ± 0.03
Neuropeptide	0.53 ± 0.04
Quorum sensing	0.41 ± 0.03
Toxicity	0.08 ± 0.03
TTCA	0.56 ± 0.01

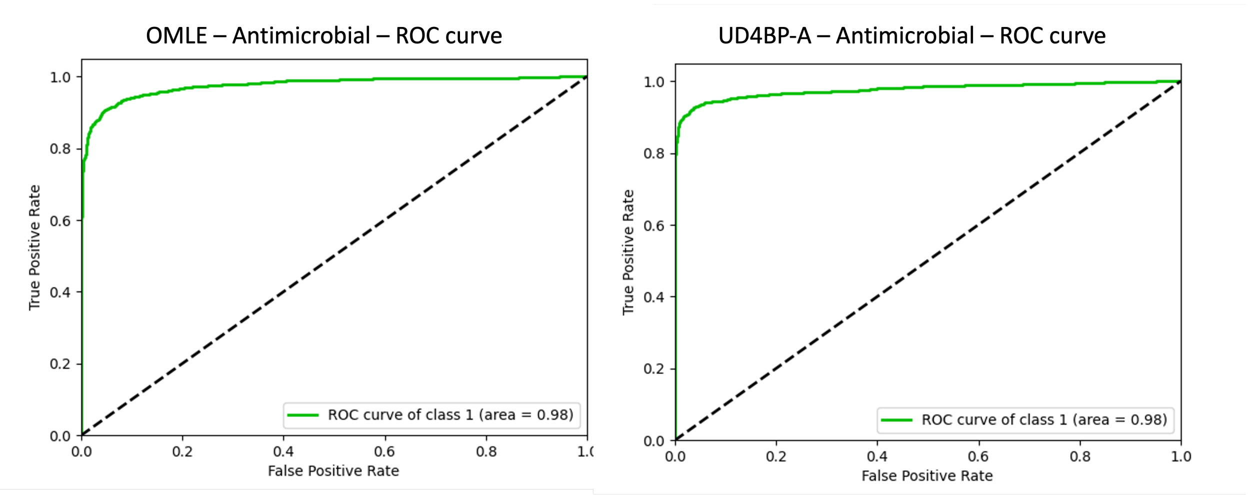

Fig. 18. ROC curves of the original Antibacterial dataset. OMLE (left) and UniDL4BioPep (right).

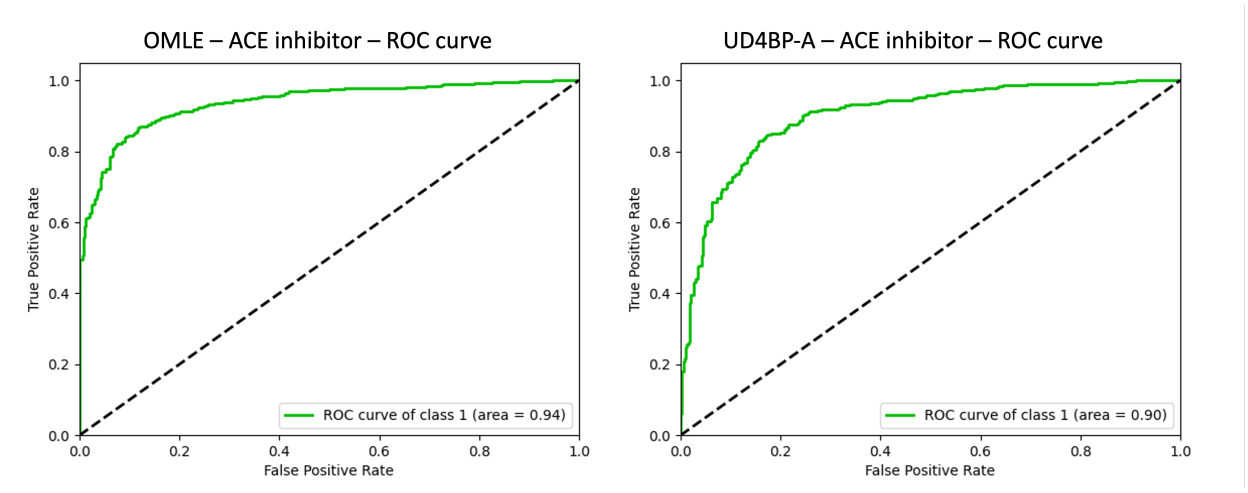

Fig. 19. ROC curves of the original ACE inhibitor dataset. OMLE (left) and UniDL4BioPep (right).

Table 8. Alternative metrics for the comparison of training strategies on original datasets. Errors represent the standard error of the mean across three different runs. ACC: Accuracy; MCC: Mathew's correlation coeficient; AUROC: Area Under the ROC curve; F1: F1 score; BBBC: Brain-blood barrier crossing; TTCA: Tumor T-cell antigens; OMLE: Optimised Machine Learning Ensemble; UDL4BP-A: UnidDLBioPep-A. For the reference of the handcrafted models, see Table

Dataset	Metric	OMLE	UDL4BP-A	Handcrafted models
Antibacterial	ACC	0.92803 ± 0.0009	0.9427 ± 0.0008	0.935
	MCC	0.858 ± 0.001	0.887 ± 0.001	0.870
	AUROC	0.9770 ± 0.0005	0.9766 ± 0.0004	0.975
	F1	0.941 ± 0.001	0.677 ± 0.002	NA
ACE inhibitor	ACC	0.855 ± 0.007	0.9280 ± 0.0009	0.883
	MCC	0.71 ± 0.01	0.67 ± 0.01	0.767
	AUROC	0.922 ± 0.008	0.911 ± 0.005	0.951
	F1	0.856 ± 0.007	0.83 ± 0.01	NA
Anticancer 1	ACC	0.717 ± 0.009	0.749 ± 0.007	0.825
	MCC	0.43 ± 0.02	0.50 ± 0.01	0.646
	AUROC	0.800 ± 0.002	0.8106 ± 0.0006	0.812
	F1	0.714 ± 0.009	0.751 ± 0.006	NA
Anticancer 2	ACC	0.939 ± 0.002	0.841 ± 0.003	0.9201
	MCC	0.879 ± 0.005	0.883 ± 0.005	0.84
	AUROC	0.9685 ± 0.0005	0.969 ± 0.001	NA
	F1	0.937 ± 0.02	0.939 ± 0.003	NA
Antifungal	ACC	0.947 ± 0.002	0.947 ± 0.002	0.942
	MCC	0.895 ± 0.004	0.894 ± 0.005	0.884
	AUROC	0.991 ± 0.002	0.9875 ± 0.0001	0.988
	F1	0.944 ± 0.002	0.946 ± 0.002	NA
Antimalarial 1	ACC	0.980 ± 0.001	0.975 ± 0.004	0.978
	MCC	0.78 ± 0.03	0.82 ± 0.01	0.776
	AUROC	0.955 ± 0.003	0.935 ± 0.003	0.82
	F1	0.828 ± 0.009	0.79 ± 0.03	NA
Antimalarial 2	ACC	0.98770 ± 0.00001	0.973 ± 0.004	0.957
	MCC	0.95660 ± 0.00001	0.91 ± 0.01	0.834
	AUROC	0.997 ± 0.001	0.993 ± 0.001	0.903
	F1	0.96300 ± 0.00001	0.92 ± 0.01	NA
Antimicrobial	ACC	0.954 ± 0.001	0.9667 ± 0.007	NA
	MCC	0.887 ± 0.003	0.919 ± 0.002	NA
	AUROC	0.9860 ± 0.0008	0.9895 ± 0.0004	NA
	F1	0.919 ± 0.002	0.942 ± 0.001	NA
Antioxidant	ACC	0.84 ± 0.01	0.831 ± 0.004	NA
	MCC	0.67 ± 0.02	0.657 ± 0.08	0.48
	AUROC	0.897 ± 0.002	0.880 ± 0.07	0.79
1.	F1	0.814 ± 0.008	0.809 ± 0.005	NA
Antiparasitic	ACC	0.757 ± 0.01	0.754 ± 0.01	0.880
	MCC	0.56 ± 0.02	0.55 ± 0.02	0.776
	AUROC	0.930 ± 0.004	0.931 ± 0.006	0.922
	F1	0.70 ± 0.03	0.70 ± 0.02	0.891
Antiviral	ACC	0.828 ± 0.005	0.835 ± 0.001	0.828
	MCC	0.659 ± 0.009	0.673 ± 0.004	0.662
	AUROC	0.898 ± 0.005	0.9083 ± 0.0007	0.896
	F1	0.821 ± 0.007	0.829 ± 0.005	NA
BBBC	ACC	0.80 ± 0.02	0.78950 ± 0.00001	0.7895
	MCC	0.60 ± 0.05	0.58220 ± 0.00001	0.6102
	AUROC	0.907 ± 0.008	0.85 ± 0.02	0.7895
	F1	0.79 ± 0.03	0.77780 ± 0.00001	0.7500
DPPIV inhibitor	ACC	0.83 ± 0.02	0.812 ± 0.004	0.797
	MCC	0.67 ± 0.04	0.624 ± 0.009	0.594
	AUROC	0.927 ± 0.002	0.911 ± 0.003	0.847
	F1	0.83 ± 0.02	0.811 ± 0.004	NA
Anti-MRSA	ACC	0.998 ± 0.002	0.988 ± 0.004	0.960
	MCC	0.993 ± 0.007	0.96 ± 0.02	0.848
	AUROC	1.00000 ± 0.00001	0.9994 ± 0.0002	0.986
	F1	0.994 ± 0.006	0.96 ± 0.01	NA
Neuropeptide	ACC	0.850 ± 0.002	0.888 ± 0.002	0.936
	MCC	0.705 ± 0.003	0.776 ± 0.004	0.875
	AUROC	0.937 ± 0.001	0.953 ± 0.001	0.988
	F1	0.858 ± 0.002	0.889 ± 0.001	NA
Quorum sensing	ACC	0.908 ± 0.008	0.917 ± 0.008	0.943
	MCC	0.82 ± 0.01	0.83 ± 0.02	0.885
	AUROC	0.967 ± 0.005	0.981 ± 0.001	0.945
	F1	0.908 ± 0.008	0.915 ± 0.008	NA
Toxicity	ACC	0.914 ± 0.004	0.913 ± 0.004	0.912 ± 0.002
	MCC	0.828 ± 0.098	0.828 ± 0.005	0.903 ± 0.004
	AUROC	0.9677 ± 0.0007	0.9708 ± 0.0006	0.976 ± 0.001
	F1	0.919 ± 0.004	0.916 ± 0.002	NA
TTCA	ACC	0.689 ± 0.009	0.72 ± 0.01	0.71
	MCC	0.33 ± 0.02	0.42 ± 0.02	0.363
	AUROC	0.70 ± 0.01	0.782 ± 0.004	0.73
	F1	0.753 ± 0.008	0.77 ± 0.01	0.756

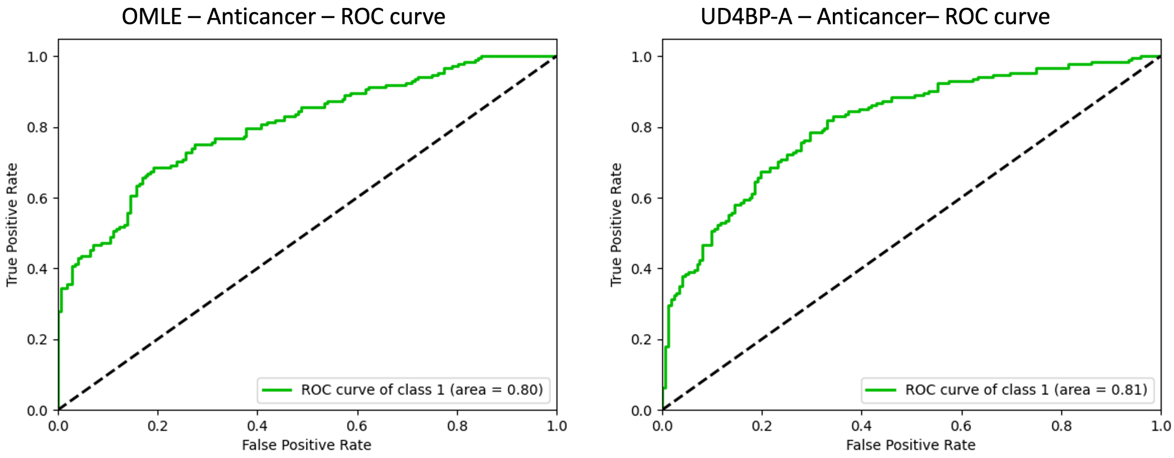

Fig. 20. ROC curves of the original Anticancer 1 dataset. OMLE (left) and UniDL4BioPep (right).

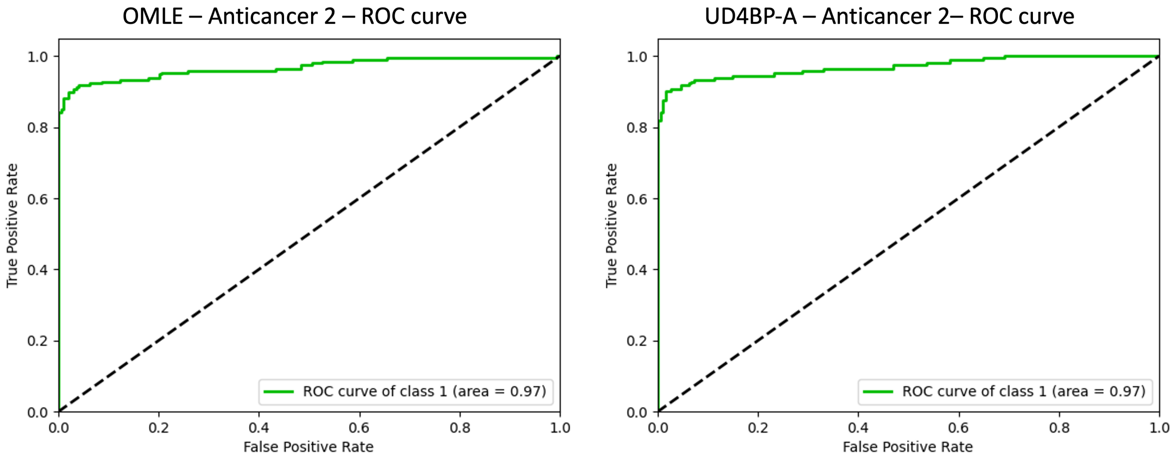

Fig. 21. ROC curves of the original Anticancer 2 dataset. OMLE (left) and UniDL4BioPep (right).

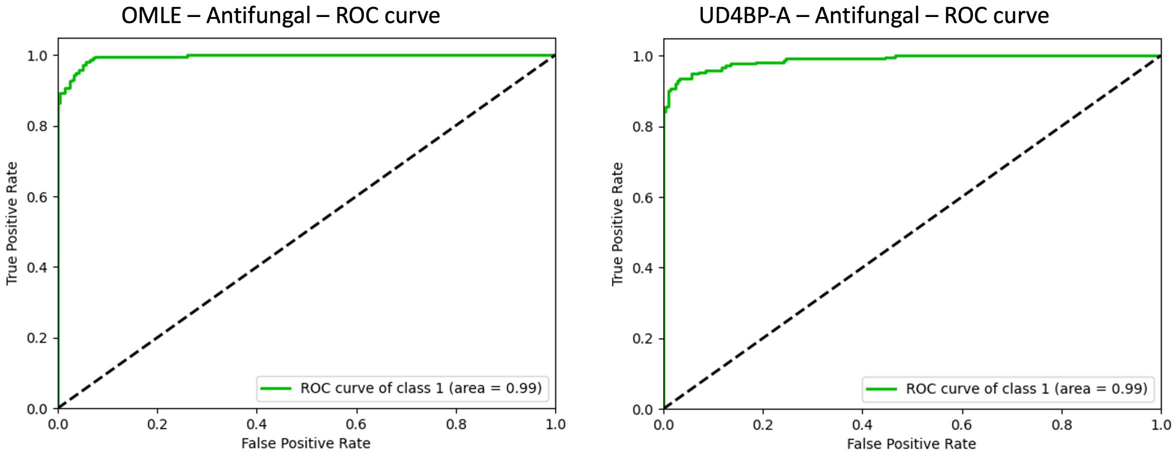

Fig. 22. ROC curves of the original Antifungal dataset. OMLE (left) and UniDL4BioPep (right).

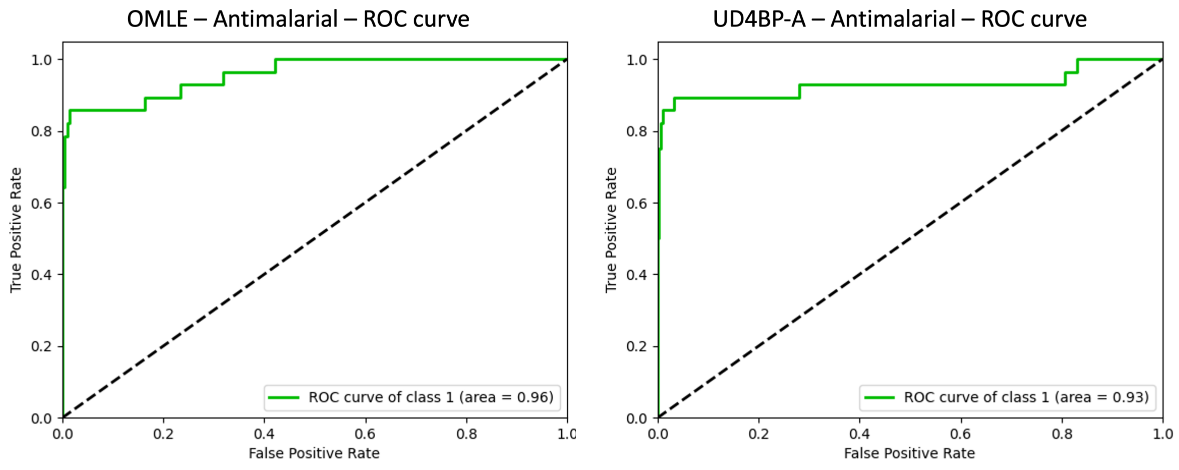

Fig. 23. ROC curves of the original Antimalarial 1 dataset. OMLE (left) and UniDL4BioPep (right).

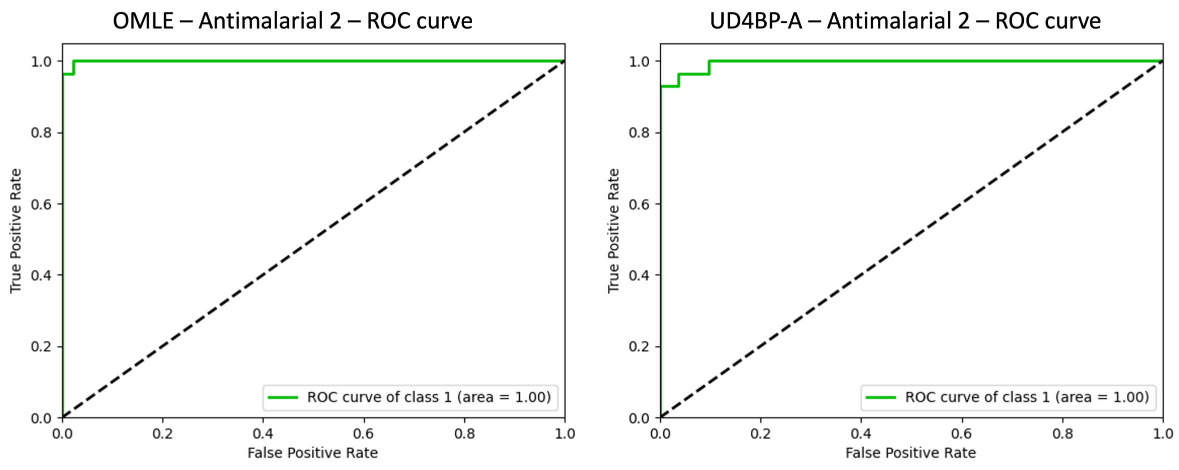

Fig. 24. ROC curves of the original Antimalarial 2 dataset. OMLE (left) and UniDL4BioPep (right).

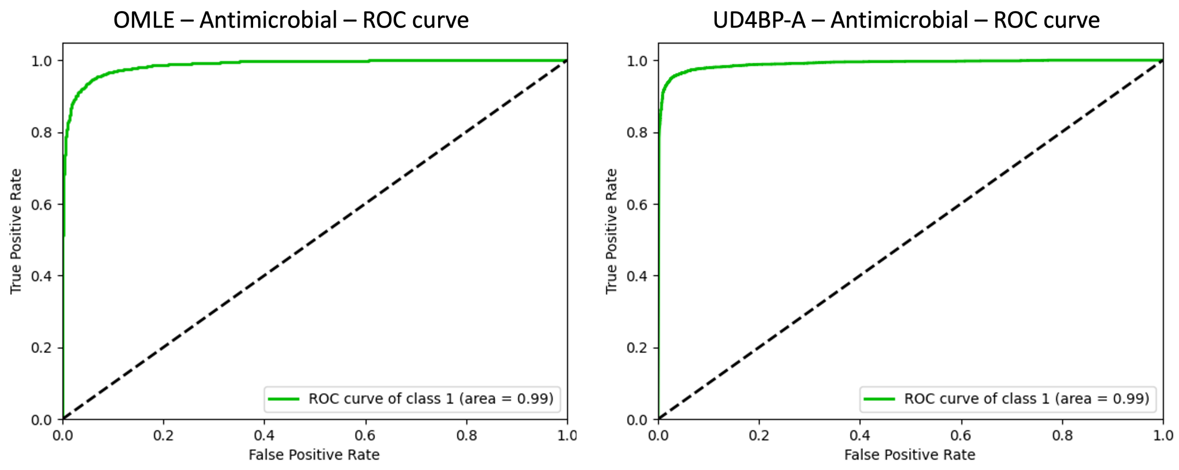

Fig. 25. ROC curves of the original Antimicrobial dataset. OMLE (left) and UniDL4BioPep (right).

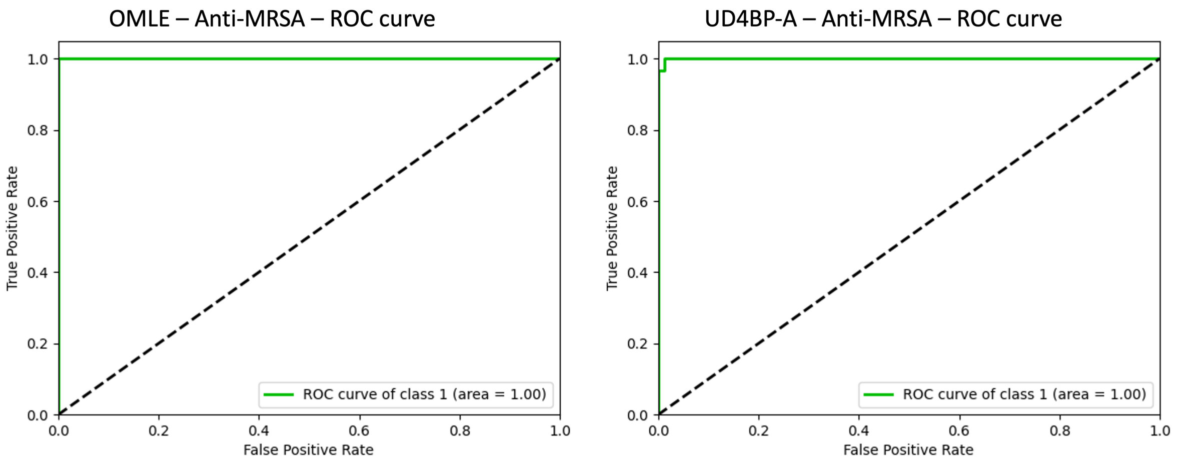

Fig. 26. ROC curves of the original Anti-MRSA dataset. OMLE (left) and UniDL4BioPep (right).

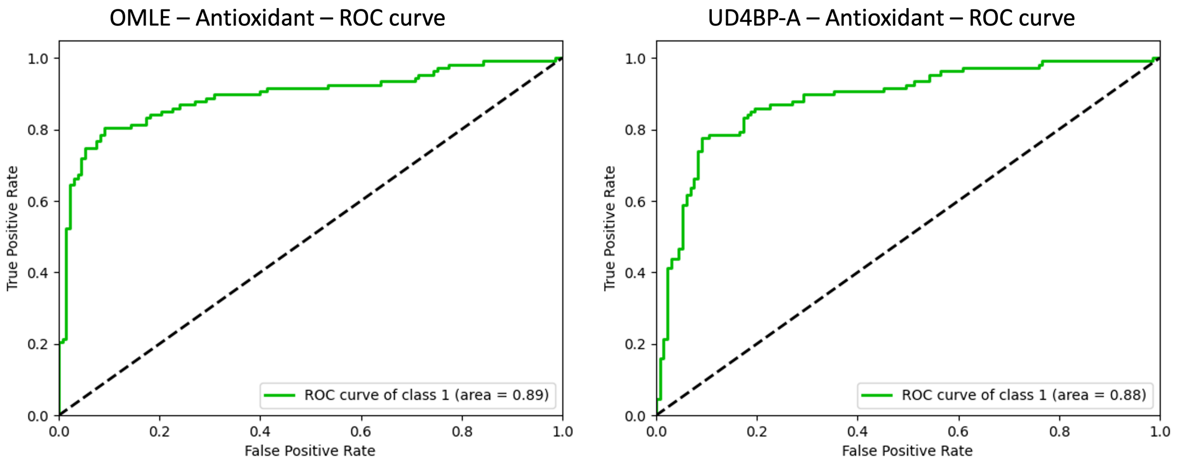

Fig. 27. ROC curves of the original Antioxidant dataset. OMLE (left) and UniDL4BioPep (right).

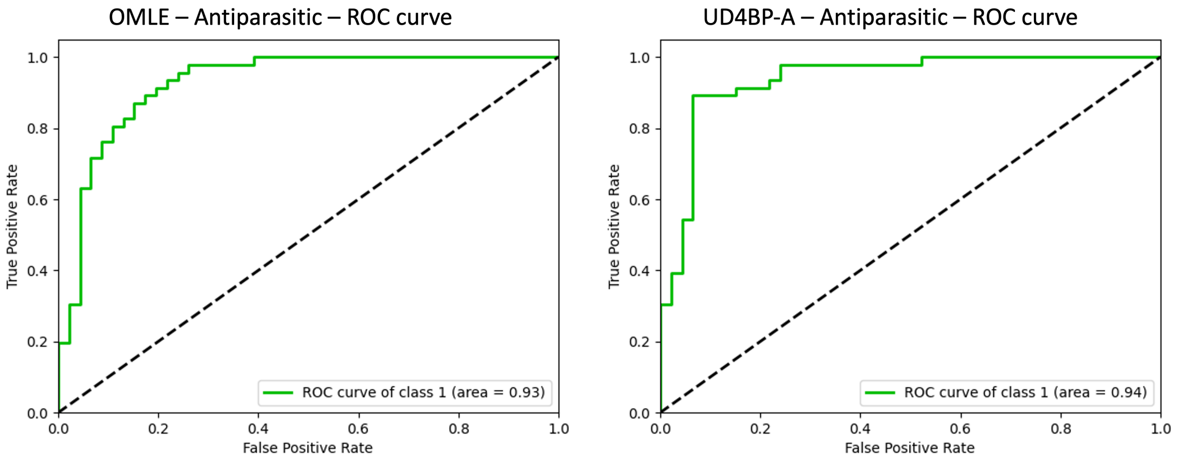

Fig. 28. ROC curves of the original Antiparasitic dataset. OMLE (left) and UniDL4BioPep (right).

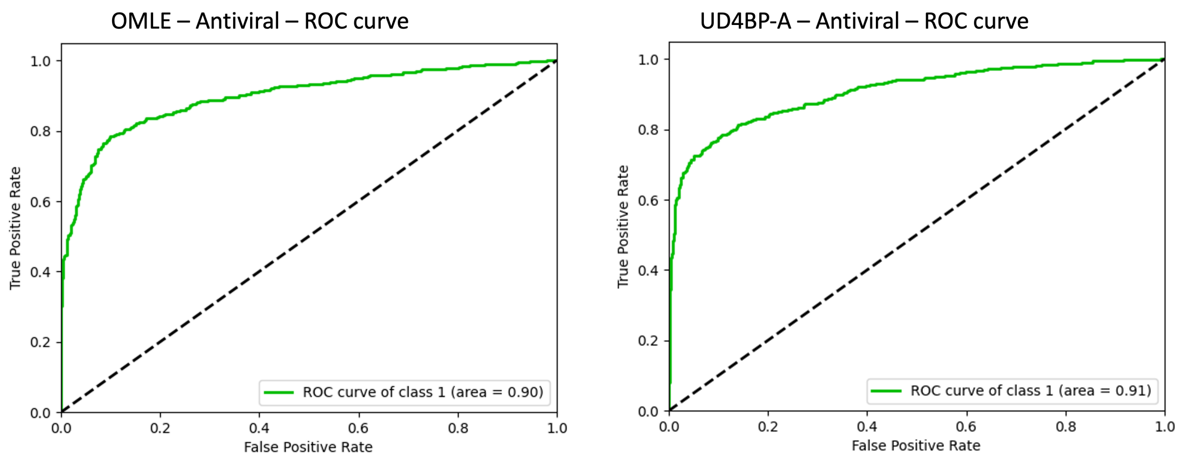

Fig. 29. ROC curves of the original Antiviral dataset. OMLE (left) and UniDL4BioPep (right).

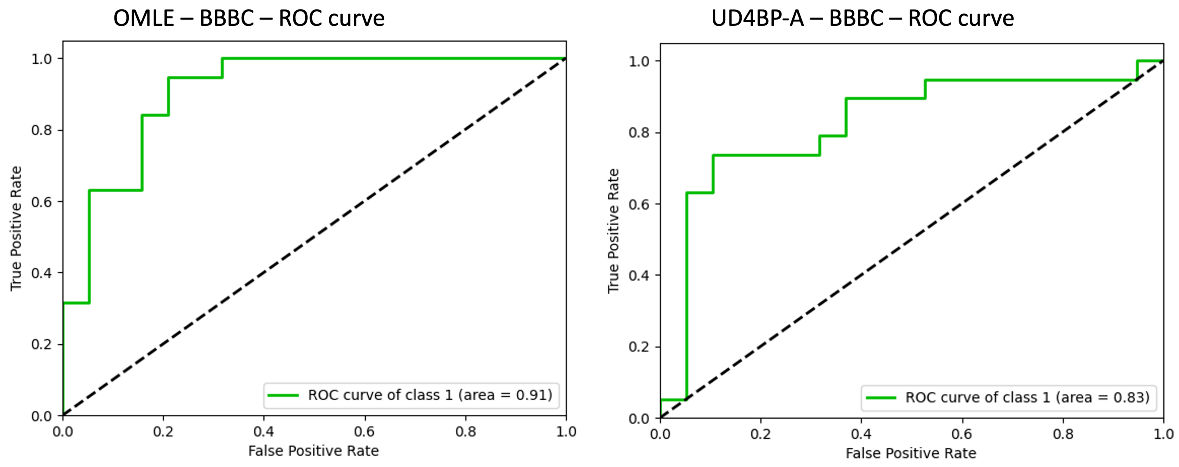

Fig. 30. ROC curves of the original Blood-brain barrier crossing dataset. OMLE (left) and UniDL4BioPep (right).

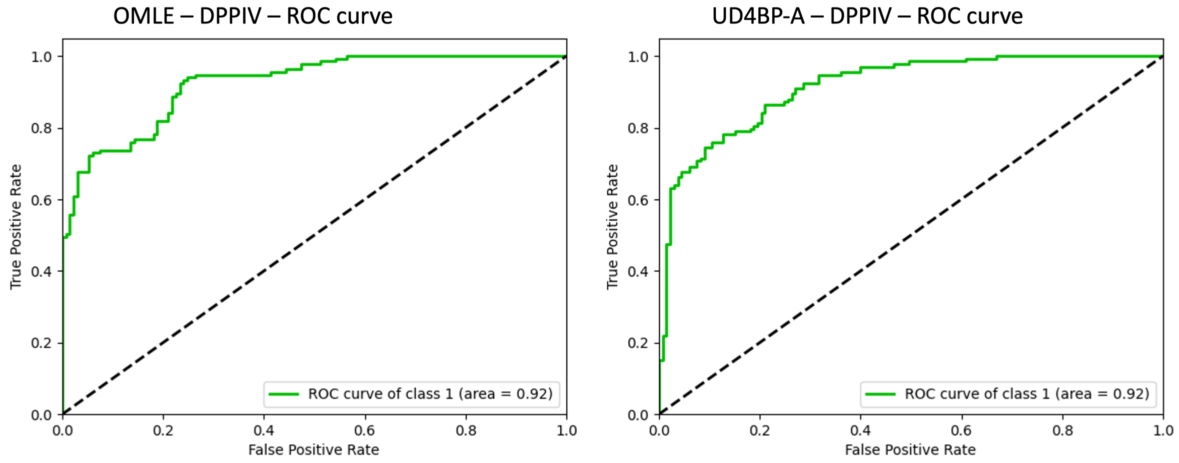

Fig. 31. ROC curves of the original DPPIV inhibitor dataset. OMLE (left) and UniDL4BioPep (right).

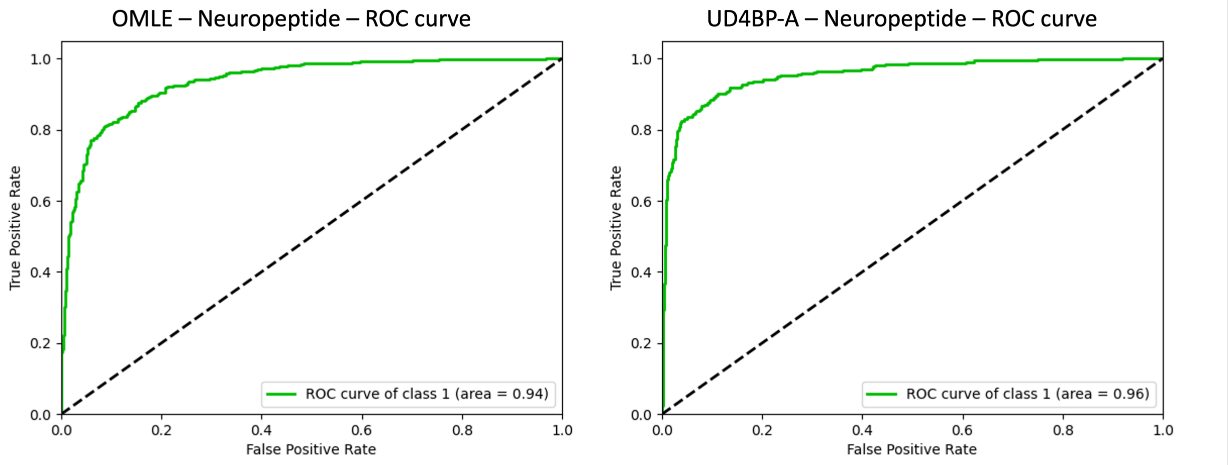

Fig. 32. ROC curves of the original Neuropeptide dataset. OMLE (left) and UniDL4BioPep (right).

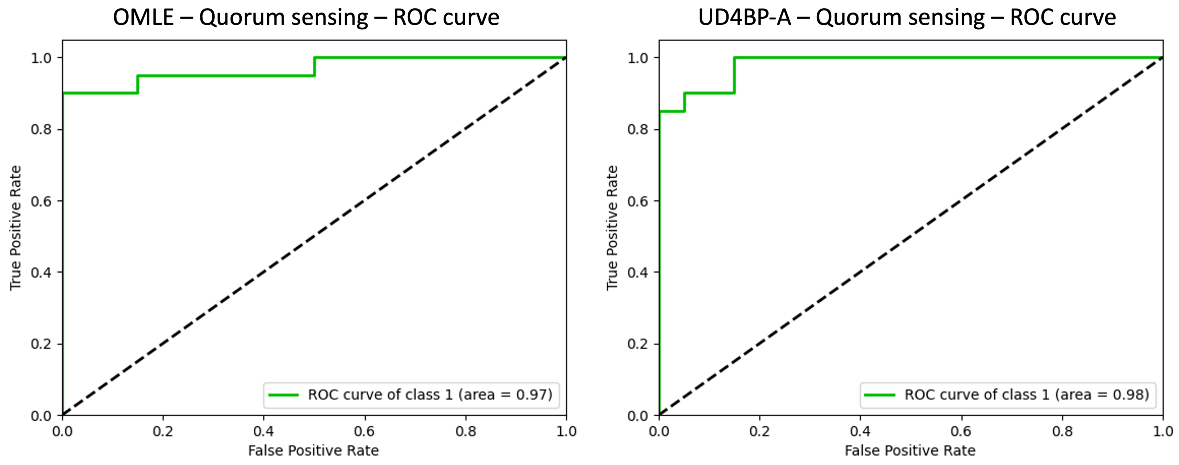

Fig. 33. ROC curves of the original Quorum sensing dataset. OMLE (left) and UniDL4BioPep (right).

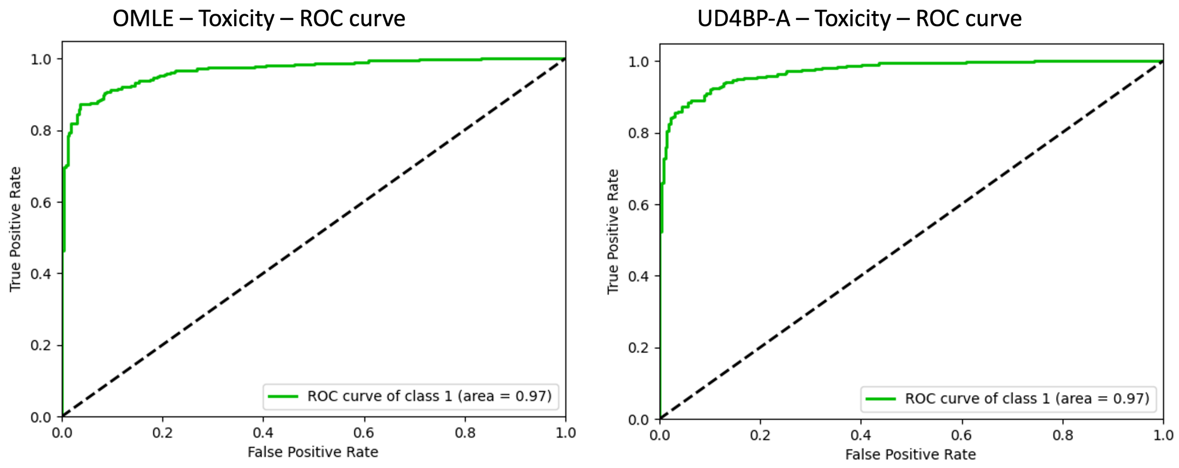

Fig. 34. ROC curves of the original Toxicity dataset. OMLE (left) and UniDL4BioPep (right).

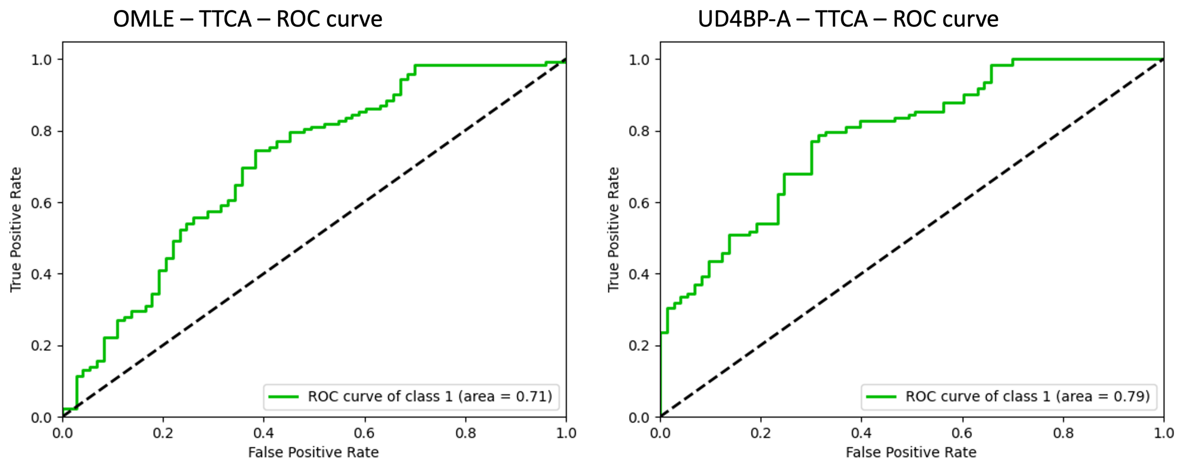

Fig. 35. ROC curves of the original Tumor T-cell antigen dataset. OMLE (left) and UniDL4BioPep (right).

Table 9. Metrics for the evaluation of training strategies in the new datasets. Errors represent the standard error of the mean across three different runs. All values correspond to Matthew's Correlation Coefficient; NegSearch: Dataset with new negative peptides; HP: Homology-based dataset partitioning module; MCC: Mathew's correlation coeficient; BBBC: Brain-blood barrier crossing; TTCA: Tumor T-cell antigens.

Dataset	OMLE NegSearch	UDL4BP-A NegSearch	OMLE NegSearch+HP	UDL4BP NegSearch+HP
Antibacterial	0.663 ± 0.004	0.656 ± 0.004	0.45 ± 0.01	0.42 ± 0.02
ACE inhibitor	0.547 ± 0.004	0.578 ± 0.004	0.57 ± 0.01	0.61 ± 0.03
Anticancer	0.547 ± 0.004	0.64 ± 0.03	0.33 ± 0.01	0.30 ± 0.02
Antifungal	0.78 ± 0.02	0.76 ± 0.02	0.20 ± 0.02	0.26 ± 0.02
Antimalarial	0.51 ± 0.08	0.41 ± 0.08	0.39 ± 0.06	0.33 ± 0.04
Antimicrobial	0.774 ± 0.004	0.749 ± 0.004	0.292 ± 0.009	0.32 ± 0.01
Antioxidant	0.31 ± 0.04	0.34 ± 0.04	0.26 ± 0.06	0.33 ± 0.05
Antiparasitic	0.58 ± 0.07	0.51 ± 0.07	0.41 ± 0.07	0.40 ± 0.06
Antiviral	0.720 ± 0.003	0.725 ± 0.003	0.520 ± 0.009	0.49 ± 0.08
BBBC	0.13 ± 0.09	0.28 ± 0.09	0.08 ± 0.08	0.19 ± 0.06
DPPIV inhibitor	0.494 ± 0.007	0.562 ± 0.007	0.47 ± 0.03	0.56 ± 0.04
Anti-MRSA	0.651 ± 0.01	0.74 ± 0.01	0.41 ± 0.09	0.49 ± 0.03
Neuropeptide	0.701 ± 0.007	0.722 ± 0.006	0.64 ± 0.01	0.708 ± 0.008
Quorum sensing	0.70 ± 0.04	0.72 ± 0.04	0.65 ± 0.08	0.68 ± 0.01
Toxicity	0.65 ± 0.03	0.64 ± 0.03	0.26 ± 0.006	0.39 ± 0.01
TTCA	0.75 ± 0.02	0.77 ± 0.02	0.74 ± 0.02	0.80 ± 0.02

G. AutoPeptideML

Recommendations for using AutoPeptideML and reporting its results

This section explores how the structure of the outputs from AutoPeptideML facilitates compliance with DOME guidelines (Walsh et al., 2021), nevertheless, it is important to note that no system can fully avoid its misuse or abuse and the ultimate responsibility of following proper guidelines and accurately reporting the results lies in the final users.

- **Data:** The algorithm ensures independence between the optimisation (training) and evaluation (test) sets. The hyperparameter optimisation and model selection, which can be considered as meta-optimisation strategies, relies on n-fold cross-validation and maintains the independence of the testing set. Further, the constraints upon the algorithm in the web-server application impedes malpractices like the manual curation of parameters to meta-optimise the results in the independent test sets.

  The datasets generated during the automatic search for negative samples, the train/test partitions, and the n train/validation folds are included in the ZIP-compressed output file, thus making their release and sharing easy. The automatic search for negatives is also compliant with the recommendation that the distribution of the data is representative of the domain in which the model is going to be applied. The use of random seeds for any stochastic process improves the reproducibility when the same exact datasets are used, thus guaranteeing that different runs will produce similar results.
- **Optimisation:** Metrics for each fold in cross-validation are provided alongside the final evaluation metrics of the model so that train versus test error can be calculated as a measure of possible under- or over-fitting. The hyper-parameter configurations of the final models are included in the output file and are therefore easy to share.
- **Model:** PLMs are not directly explainable and it follows that models built on top of their representations are thus not explainable.
- **Evaluation:** Models are evaluated with a wide array of metrics and a PDF summary of the main model performance plots and evaluation metrics is provided with a guide on how to interpret them depending on different application contexts meant for researchers that are not familiar with ML concepts. Most common problems when analysing evaluation metrics arise when working with imbalanced testing datasets, the automatic dataset construction module bypasses this problem by generating balanced datasets.

Output

The output for AutoPeptideML is generated in a ZIP-compressed directory with the following subdirectories:

- `apml_config.json`: File describing the configuration settings used to run AutoPeptideML. It allows the reproduction of experiments as it also contains the seed for the pseudo-random number generator for all stochastic processes.
- `best_configs`: It is a subdirectory containing the best combination of hyperparameters found for all models. It will contain as many configuration files as separate hyperparameter searches run. By default, it will be three.
- `ensemble`: It is a subdirectory that contains the trained models.
- `evaluation_data`: It is a subdirectory that contains two files: 1) `cross-validation.csv`, which is a CSV file with the model performance metrics during hyperparameter optimisation; 2) `test_scores.csv`, which is a CSV file with the final model performance metrics from the evaluation against the testing set.
- `figures`: It is a subdirectory that contains different interesting figures for analysing model performance including: ROC curve, calibration curve, confusion matrix, and precision-recall curve.
- `folds` It is a subdirectory that contains the n cross-validation folds, allows for reproducing the experiments.
- `splits` It is a subdirectory that contains the train and test partitions, allows for reproducing the evaluation.
- `summary.pdf`: It is an automatically generated summary of the evaluation metrics and all the figures in the `figures` subdirectory and guidance on how to interpret both metrics and figures.
