# Supplementary figures and images for: AutoPeptideML: a study on how to build more trustworthy peptide bioactivity predictors
Source: Bioinformatics. 2024 Sep 18;40(9):btae555. doi: 10.1093/bioinformatics/btae555 (PMC11438549; doi:10.1093/bioinformatics/btae555)

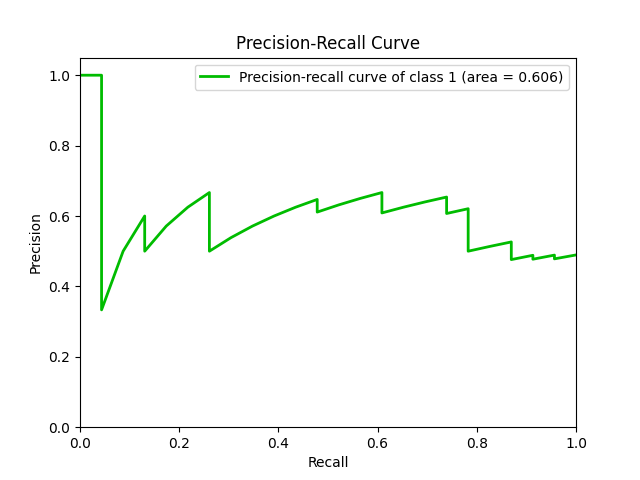

Supplement: btae555_Supplementary_Data [file btae555_supplementary_data.zip › precision_recall_curve.png]

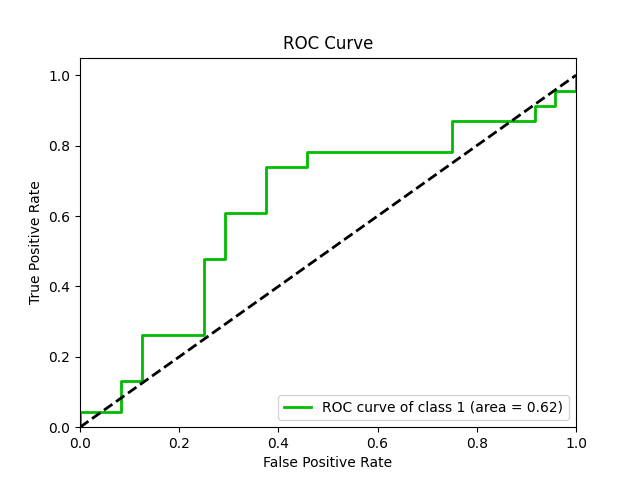

Supplement: btae555_Supplementary_Data [file btae555_supplementary_data.zip › roc_curve.png]

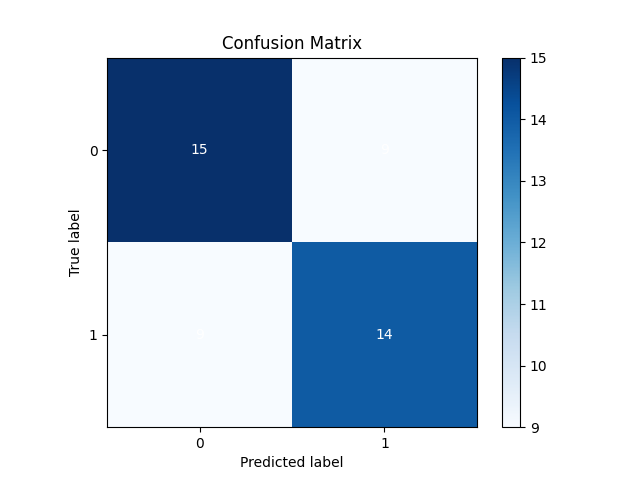

Supplement: btae555_Supplementary_Data [file btae555_supplementary_data.zip › confusion_matrix.png]

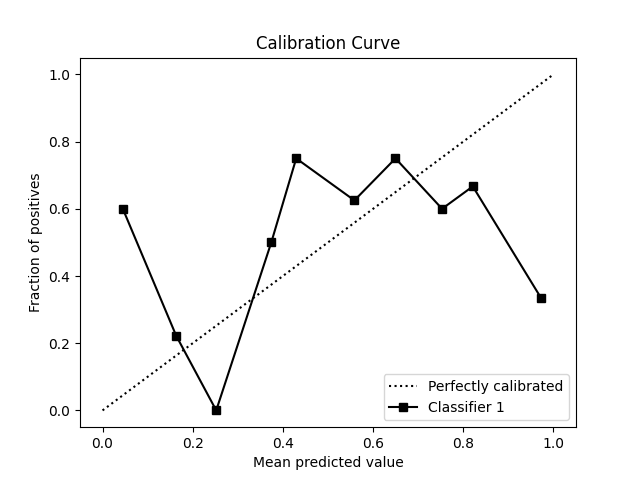

Supplement: btae555_Supplementary_Data [file btae555_supplementary_data.zip › calibration_curve.png]
